# Integrated transcriptomics, proteomics, and functional analysis to characterize the tissue‐specific small extracellular vesicle network of breast cancer

**DOI:** 10.1002/mco2.433

**Published:** 2023-12-03

**Authors:** Lesang Shen, Huanhuan Huang, Zichen Wei, Wuzhen Chen, Jiaxin Li, Yao Yao, Jun Zhou, Jian Liu, Shanshan Sun, Wenjie Xia, Ting Zhang, Xiuyan Yu, Jun Shen, Weilan Wang, Jingxin Jiang, Jian Huang, Ming Jiang, Chao Ni

**Affiliations:** ^1^ Department of Breast Surgery Second Affiliated Hospital, Zhejiang University Hangzhou China; ^2^ Key Laboratory of Tumor Microenvironment and Immune Therapy of Zhejiang Province Second Affiliated Hospital, Zhejiang University Hangzhou China; ^3^ Cancer Center Zhejiang University Hangzhou China; ^4^ Center for Genetic Medicine The Fourth Affiliated Hospital, Zhejiang University School of Medicine Hangzhou China; ^5^ Department of Anesthesiology Taihe Hospital Hubei University of Medicine Shiyan China; ^6^ Department of Breast Surgery Affiliated Hangzhou First People's Hospital Zhejiang University Hangzhou China; ^7^ Department of Breast Surgery Zhejiang Provincial People's Hospital Hangzhou China; ^8^ Department of Radiation Oncology Second Affiliated Hospital Zhejiang University Hangzhou China; ^9^ Department of Surgical Oncology Sir Run Run Shaw Hospital, Zhejiang University Hangzhou China; ^10^ Department of Breast Surgery Changxing People's Hospital Huzhou China; ^11^ Zhejiang Provincial Key Laboratory of Genetic and Developmental Disorders Hangzhou China

**Keywords:** breast cancer tissues, immunoregulation, multiomics profiling, organoids, small extracellular vesicles

## Abstract

Small extracellular vesicles (sEVs) are essential mediators of intercellular communication within the tumor microenvironment (TME). Although the biological features of sEVs have been characterized based on in vitro culture models, recent evidence indicates significant differences between sEVs derived from tissue and those derived from in vitro models in terms of both content and biological function. However, comprehensive comparisons and functional analyses are still limited. Here, we collected sEVs from breast cancer tissues (T‐sEVs), paired normal tissues (N‐sEVs), corresponding plasma (B‐sEVs), and tumor organoids (O‐sEVs) to characterize their transcriptomic and proteomic profiles. We identified the actual cancer‐specific sEV signatures characterized by enriched cell adhesion and immunomodulatory molecules. Furthermore, we revealed the significant contribution of cancer‐associated fibroblasts in the sEV network within the TME. In vitro model‐derived sEVs did not entirely inherit the extracellular matrix‐ and immunity regulation‐related features of T‐sEVs. Also, we demonstrated the greater immunostimulatory ability of T‐sEVs on macrophages and CD8+ T cells compared to O‐sEVs. Moreover, certain sEV biomarkers derived from noncancer cells in the circulation exhibited promising diagnostic potential. This study provides valuable insights into the functional characteristics of tumor tissue‐derived sEVs, highlighting their potential as diagnostic markers and therapeutic agents for breast cancer.

## Graphical abstract

BC‐specific sEV network was characterized by enriched cell adhesion and immunomodulatory molecules, in which the contribution of CAFs was highlighted. BC tissue‐derived sEVs presented a stronger immunostimulatory function than in vitro models. Moreover, some noncancer cell‐derived sEV biomarkers in circulation have great diagnostic potential.

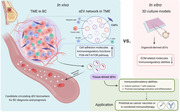

## 1 INTRODUCTION

Small extracellular vesicles (sEVs) are nanometric vesicles (less than 200 nm in diameter) enveloped by a lipid bilayer and formed in vesicular bodies with the endosomal network.^1^, ^2^ sEVs can be released by almost all cell types and play critical roles in mediating intercellular communication by exchanging biomolecular cargoes, which consist of proteins, lipids, and genomic contents.^3^, ^4^ To date, increasing evidence has proven that the contribution of sEVs in cancer is not limited to influencing tumor progression directly but also participates in immune regulation and is valuable for cancer diagnosis and recurrence surveillance.^4^, ^5^

Previous studies primarily focused on sEVs derived from cell line supernatants or body fluids.^4^, ^6^, ^7^ Recent evidence suggests distinguishing patterns of sEVs in the tumor microenvironment (TME).^8^, ^9^ The entire sEV network is affected by a variety of factors within the TME, such as oxygen tension and acidity.^10^, ^11^ With the optimization of tissue sEV isolation protocols, tissue‐derived sEVs have recently been isolated and characterized in several solid tumors.^12^, ^13^, ^14^, ^15^ However, the report on breast cancer (BC) is still lacking. Despite the complex sEV cargoes, no previous studies have simultaneously presented a comprehensive portrait of tissue‐derived sEVs on transcriptomic and proteomic levels. Additionally, several preclinical studies and early‐stage clinical trials have suggested the potential for applying sEVs as vaccines or boosting antitumor immunity in cancer patients.^16^, ^17^, ^18^, ^19^ Due to the importance of sEVs in normal biological function and the difficulty of enriching cancer‐specific sEVs within circulation,^20^ more in‐depth studies are needed to decipher the actual sEV network within TME.

sEVs from different cell populations were selectively secreted into the circulation,^21^, ^22^ implying that body liquid‐derived sEVs may not be valid markers for actual human disease. Moreover, the use of 3D culture systems recently, such as organoids, provides a valuable tool to study sEVs in a more physiological context, as organoids can inherit the genomic and molecular characteristics of the donor tumors.^23^, ^24^, ^25^ To date, there have been no studies that simultaneously compare sEVs derived from tissues, in vitro models and the circulation. It is also unknown whether in vitro models adequately represent the characteristics of sEVs derived from actual tumor tissues.

In this study, we aim to bridge the gap in knowledge by comprehensively characterizing the transcriptomic and proteomic profiles of sEVs derived from BC tissues (T‐sEVs), paired normal tissues (N‐sEVs), corresponding plasma (B‐sEVs), and tumor organoids (O‐sEVs). Through multiomics profiling and functional assessments, we identified BC‐specific sEV cargoes and revealed the predominant contribution of cancer‐associated fibroblasts (CAFs) to the tissue sEV network. Moreover, our study demonstrated the superior immunostimulatory ability of T‐sEVs over O‐sEVs. The diagnostic potential of circulating sEV biomarkers from the TME were also explored here. We believe our work represents a significant advancement in unraveling the heterogeneity of sEVs from different sources.

## 2 RESULTS

### 2.1 Isolation of sEVs and identification of their transcriptomic and proteomics landscape

**FIGURE 1:**
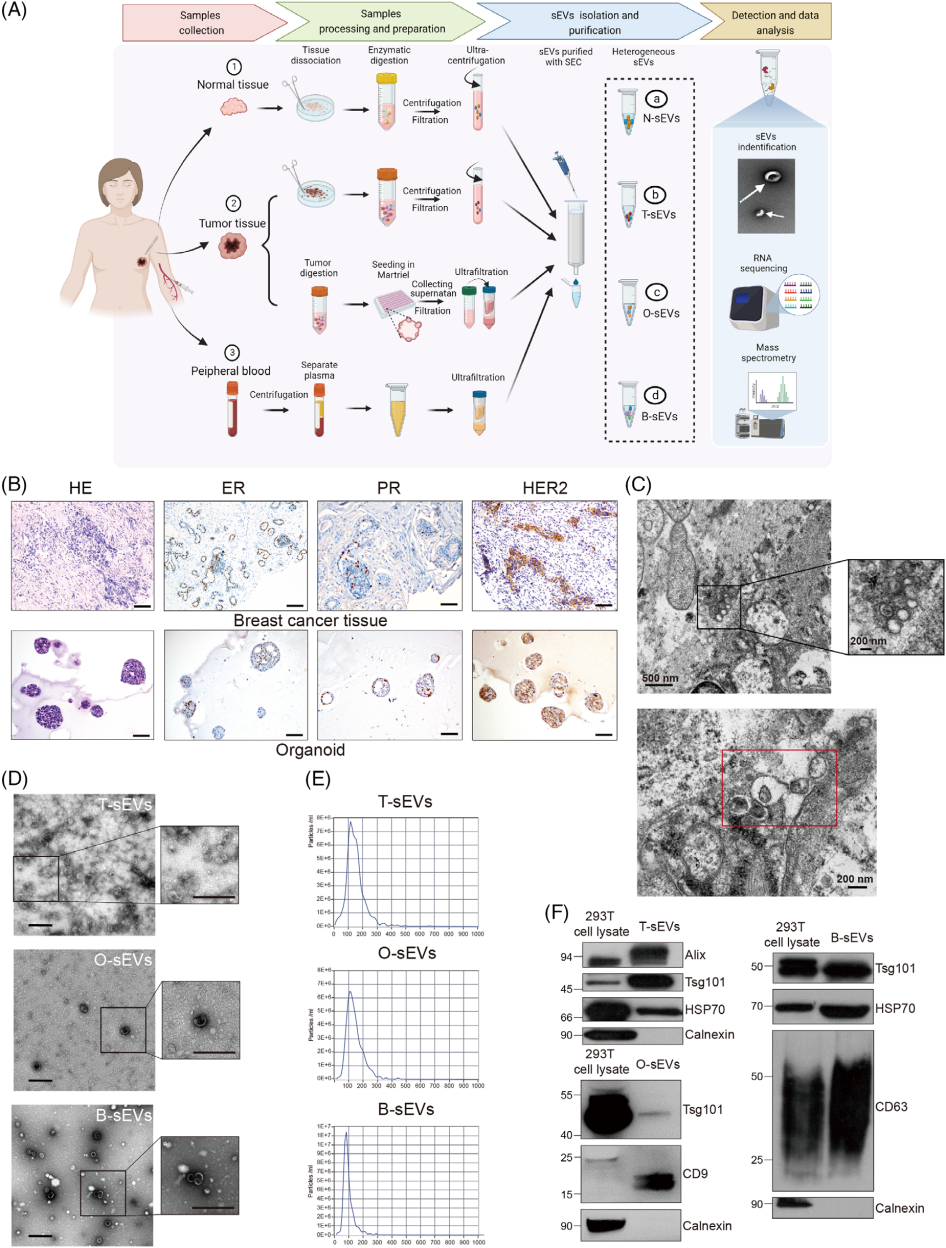
Isolation and characterization of small extracellular vesicles (sEVs) derived from various models of breast cancer (BC). (A) Schematic diagram of the collection of sEVs from fresh tissues and plasma of BC patients and tumor organoid culture. (B) Representative HE and immunohistochemical staining for estrogen receptor (ER), progesterone receptor (PR), and human epidermal growth factor receptor‐2 (HER2) expression in BC tissue and paired organoids from one patient with triple‐positive BC. Scale bars, 100 μm. (C) High magnification pictures showed numerous sEVs in the interstitial space of breast cancer tissues. Characterization of multi‐model‐derived sEVs using transmission electron microscopy (D) and nanoparticle tracking analysis (E). Scale bars, 0.5 μm. (F) Representative western blot image of staining with Alix, Tsg101, HSP70, CD9, CD63, and Calnexin in multimodel‐derived sEVs. Cell lysates were included as controls.

We conducted an extensive study according to the scheme presented in Figure 1A. The basic information of the samples used for successful sEV extraction is listed in Table S1. The tumor organoids were successfully established, which was demonstrated by morphology and immunohistochemistry staining (estrogen receptor [ER], progesterone receptor [PR], and human epidermal growth factor receptor‐2 [HER2]) compared to the primary pathological patterns (Figure 1B). The existence of sEVs in the interstitial space of BC tissues was visualized by transmission electron microscopy (TEM) (Figure 1C). Next, we tried to isolate T‐sEVs and N‐sEVs based on the isolation strategy of ultracentrifugation (UC) combined with size exclusion chromatography (SEC). The morphology and size of sEVs from various sources were identified with TEM, showing typical lipid bilayer particles ranging from 50−150 nm (Figure 1D). Nanoparticle tracking analysis (NTA) further proved that most of the particles from all sources were approximately 100 nm in diameter (Figure 1E). In addition, the western blot results revealed the expression of EV markers (Alix, Tsg101, HSP70, CD63, and CD9) in all isolated sEVs, while the endoplasmic reticulum Calnexin was detected only in cell lysates (Figure 1F).

Next, we performed transcriptomic and proteomic analyses on sEVs from all samples. The proportions of mRNA in tissue‐derived sEVs (T‐sEVs and N‐sEVs) and B‐sEVs were higher than those in O‐sEVs, while lncRNAs accounted for only a minority of long RNA profiles in all samples (Figure S1A). In addition, the number of proteins in tissue‐derived sEVs was also higher than that detected in sEVs from the other two sources (Figure S1B). Similar to previous sEV studies focusing on proteomes,^14^, ^26^ there was a moderate level of consistency among samples from the same source, and the heterogeneity could still be observed, particularly in the tissue‐derived groups (Figure S1C), which was in line with the other study[14]. Besides, the contents of sEVs derived from tissues were quite different from in vitro models, which might contribute to the change in cell populations and the microenvironment between in vivo and in vitro. In terms of the RNA profiles, significant heterogeneity was revealed among samples from different individuals. This phenomenon was consistent with a previous study by Steenbeek et al.,^27^ in which RNA expression in EVs from mice TME was explored. Therefore, the subsequent transcriptome analysis was based on individual paired samples, while proteomic analysis was grouped according to different sources.

To explore the potential biological function modulated by sEV proteins, GSVA was conducted and indicated that some cancer‐associated pathways were enriched in T‐sEVs (Figure S1D), including the PI3K‐AKT‐mTOR, DNA repair and MYC target pathways, which are commonly aberrantly activated in BC.^28^ It is worth noting that hypoxia and protein secretion pathways were prominently enriched in sEVs derived from in vivo sources. Given that the PI3K‐AKT pathway has been recognized as the most aberrantly activated signaling in BC, and have been reported regulated by sEVs to promote tumor progression,^29^ we examined the expression of phosphorylated PI3K, AKT and mTOR in different models with immunohistochemistry, and found the higher expression in tumor tissues compared with paired in vitro models (Figure S2). These results supported our bioinformatics results that T‐sEVs more likely to activate PI3K‐AKT‐mTOR pathway.

### 2.2 Identification of a cancer‐specific sEV signature by comparing T‐sEVs with N‐sEVs

**FIGURE 2:**
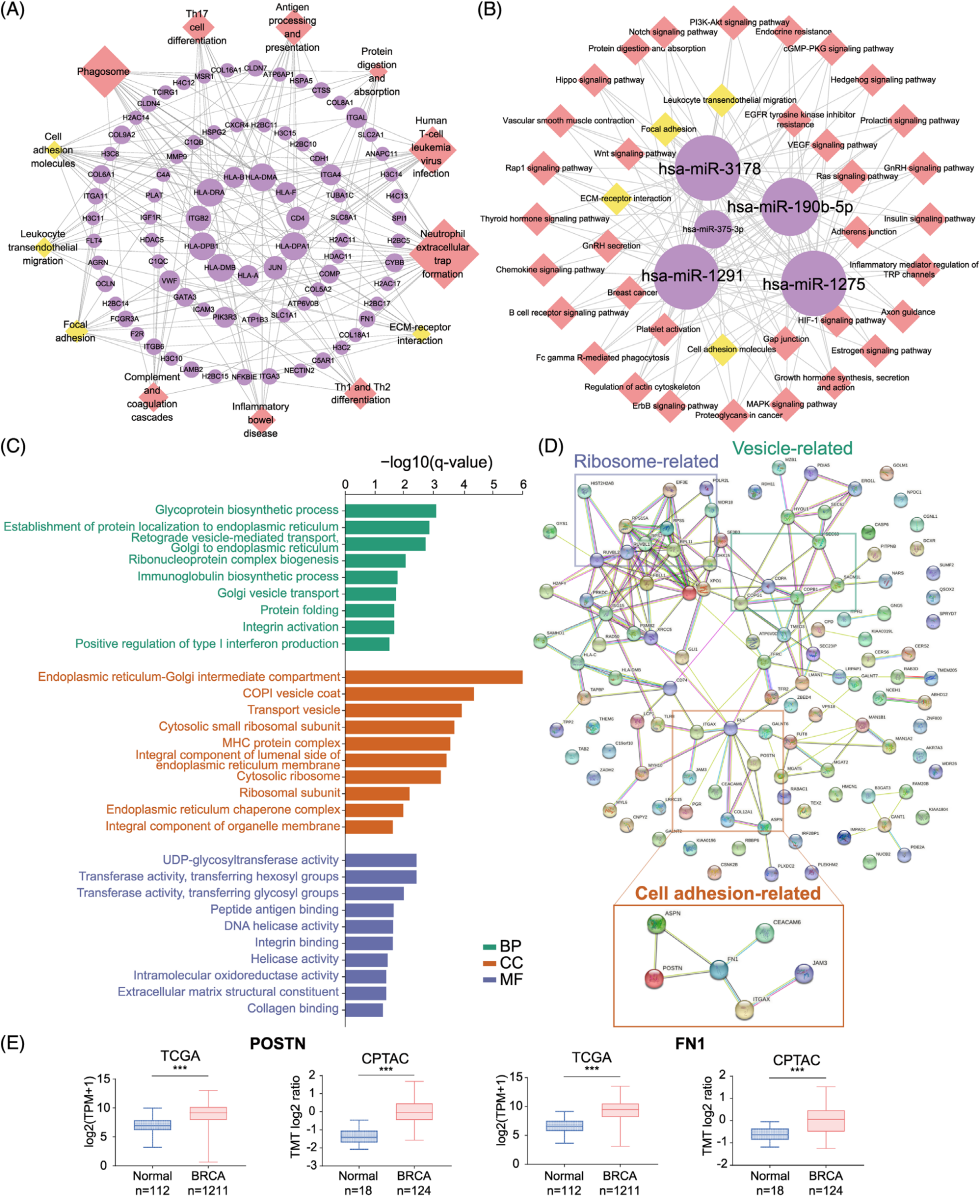
Transcriptomic and proteomic analysis of fresh tissue‐derived small extracellular vesicles (sEVs) reveals a breast cancer (BC)‐specific sEV signature. A total of 418 mRNAs and 27 microRNAs were identified as cancer‐specific sEV RNAs that were upregulated in more than half of the samples of T‐sEVs (4 of 7) when compared with paired N‐sEVs through single‐sample comparisons (fold change > 1.5 and *p* < 0.05) (gene list in Table S2). KEGG analysis was performed for these defined mRNAs and target genes of microRNAs. mRNA (A) and microRNA (B) KEGG pathway networks showing unique (red modules) and common (yellow modules) regulatory functions of cancer‐specific sEVs, which were visualized by Cytoscape software. (C) Gene ontology analysis was performed for proteins upregulated in T‐sEVs compared to N‐sEVs (fold change >4 and *p* < 0.05). The full table is shown in Table S3. (D) Protein–protein interaction network analysis of the proteins at a level >four‐fold higher in T‐sEVs than in N‐sEVs (*p* < 0.05) using STRING revealed several specific modules, including the cell adhesion‐related module, in which six extracellular matrix‐related proteins directly and indirectly interacted. (E) Transcriptomic and proteomics levels of POSTIN and FN1 were examined in normal and BC tissues based on TCGA‐BRCA and CPTAC‐BRCA data. The whiskers represent the minimum and maximum values, and the lines inside the box represent the median. A two‐tailed Mann–Whitney test was used to compare differences. ***p < 0.001.

To identify the actual cancer‐specific sEV signature, we first explored the distinct mRNA and microRNA expression patterns within T‐sEVs compared with paired N‐sEVs. 418 mRNAs and 27 microRNAs were upregulated in more than half (4 of 7) of T‐sEVs (Table S2). Among the upregulated genes, 408 mRNAs have been defined, and only five microRNAs were identified in the miRbase. These results indicated that the tissue derived sEV‐microRNA profile was greatly shared between tumor and normal tissue (microRNA: 27 of 5354, mRNA: 418 of 36173, *p* < 0.001 by chi‐square test). The cancer‐specific sEV signatures of mRNAs and microRNAs were further built into mRNA/microRNA‐pathway network analyzed by Kyoto Encyclopedia of Genes and Genomes (KEGG) (Figure 2A,B). The results suggested that cancer‐specific sEVs presented the regulating functions of immunity (leukocyte transendothelial migration) and stromal microenvironment (cell adhesion molecules and focal adhesion).^30^

In terms of the immunomodulatory function of the cancer‐specific sEV signature, the mRNAs were enriched in “neutrophil extracellular trap (NET) formation”, “Th1 and Th2 cell differentiation,” and “antigen processing and presentation”. We recently reported that tumor‐associated neutrophils promoted the lung metastasis of BC via the SIRT1‐neutrophil‐NET axis,^31^ and the Th1/Th2 differentiation imbalance and impaired antigen‐presenting cell function were also related to BC development.^32^, ^33^ On the other hand, predicted targets of upregulated microRNAs were also found to be associated with “B‐cell receptor signaling” and “platelet activation.” Except for miR‐1291, other cancer‐specific sEV microRNAs, including miR‐1275, miR‐3178, miR‐375, and miR‐190b, have been reported to be overexpressed in BC.^34^, ^35^, ^36^, ^37^ Moreover, sEV miR‐375 has been reported to facilitate tumor metastasis and chemotherapy resistance, shape immune cell phenotypes, and polarize CAFs in various cancers.^38^, ^39^, ^40^, ^41^

Next, we compared the protein profile between T‐sEVs and N‐sEVs. A total of 467 proteins were significantly upregulated and 121 proteins were significantly downregulated in T‐sEVs (Table S3). GO enrichment analysis for highly upregulated proteins in T‐sEVs (fold change >4) showed enrichment in the biological process (BP) of integrin activation and in the molecular function (MF) of integrin binding, extracellular matrix (ECM) structural constituent and collagen binding (Figure 2C). Protein‒protein interaction (PPI) network analysis identified cell adhesion‐related proteins, including ASPN, POSTN, FN1, ITGAX, JAM3, and CEACAM6 (Figure 2D). Additionally, POSTN and FN1 were highly expressed in BC tissues according to both TCGA and CPTAC databases (Figure 2E). Although previous publications have noted the involvement of these proteins in the malignant behavior of various tumors, including BC,^42^, ^43^, ^44^, ^45^, ^46^ their sEV‐dependent mechanism has not yet been reported, which might be attributed to previous sEV studies based on in vitro culture models.

### 2.3 Prediction of the cellular sources of sEVs within the TME

**FIGURE 3:**
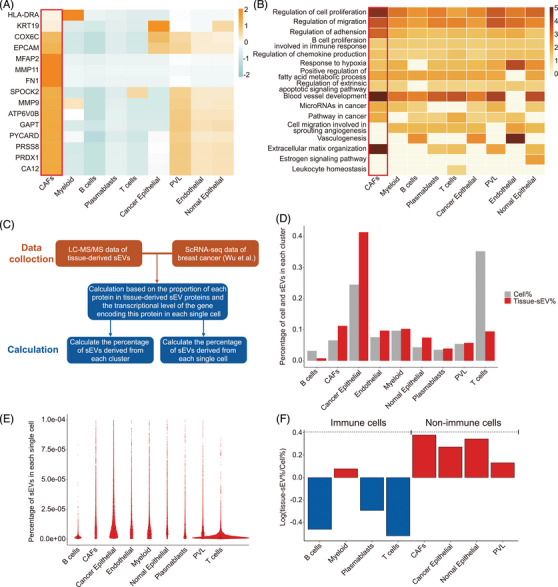
Cancer‐associated fibroblasts (CAFs) are the primary contributing cell types in the small extracellular vesicle (sEV) network within the TME. (A) Heatmap of the traced cell types sorted by the normalized cwFlod change of DEGs upregulated in T‐sEVs compared to N‐sEVs (fold change >1.5 and *p* < 0.05) (gene list in Table S4). (B) The cell‐type‐specific pathway analysis of DEGs and corresponding enriched pathways are listed in the heatmap. (C) Schematic diagram of cellular source estimation of tissue‐derived sEVs by combining our LC‐MS/MS data and public scRNA‐seq data. (D) Percentage of the number of cells in each cluster in total cells (Cell%) and percentage of the amount of sEVs from each cluster in total sEVs (Tissue‐sEV%). (E) The percentage of sEVs derived from each single cell in total sEVs is shown in violin plots. Data are presented as the median with interquartile ranges, *n* = the number of cells in each cluster. (F) The value of log(Tissue‐sEV%/Cell%) in each cell cluster, shown as the classification of immune and nonimmune cells.

Due to the complex origins of sEVs in the TME, it is still difficult to directly identify the specific cellular sources of sEVs. Here, we conducted tracking analyses to investigate the cellular sources of tissue‐derived sEVs based on sEV mRNA and proteomic profiles. Compared with N‐sEVs, we first identified 38 differentially expressed genes (DEGs) that were upregulated in T‐sEVs (Table S4). A single‐cell mapper (scMappR) was applied to determine cell types tracked for discriminating tumors and controls based on our sEV mRNA sequencing data and a scRNA‐seq database.^47^, ^48^ We then assigned the DEG‐contributed cell types, identified the cell types with the highest cwFold change, and found that CAFs were the dominant contributing cell subtype. The other cell subtypes included perivascular‐like, endothelial, and normal epithelial cells (Figure 3A). In addition, the corresponding highly enriched pathways also suggested classical biological functions of CAFs,^49^ including promoting tumor progression, vessel development, and ECM remodeling (Figure 3B).

Next, we reanalyzed the cellular sources of T‐sEVs with another strategy^50^ based on our liquid chromatography‐tandem mass spectrometry (LC‐MS/MS) data and public scRNA‐seq data (Figure 3C). Integrative analysis indicated that cancer epithelial cells accounted for the most source of sEVs, followed by CAFs (Figure 3D). However, when calculating the ability of single cells to release sEVs, we found a superior level of CAFs compared with cancer epithelial cells (Figure 3E). Moreover, nonimmune cells were more capable of releasing sEVs than immune cells (Figure 3F). Together, these results suggested the importance of non‐cancer cell populations, especially CAFs, in the sEV network within the TME.

### 2.4 sEV molecule patterns lost in in vitro culture models versus T‐sEVs

**FIGURE 4:**
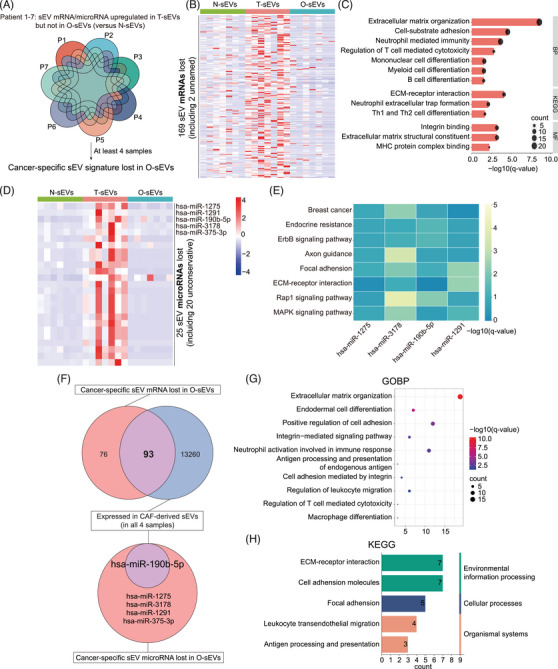
Cancer‐specific small extracellular vesicle (sEV) signature lost in organoid culture. (A) A total of seven pairs of completely paired samples (with N‐sEVs, T‐sEVs, and O‐sEVs) were included. For each patient, the upregulated sEV molecules in T‐sEVs compared to N‐sEVs (fold change >1.5 and *p* < 0.05), which were not upregulated in O‐sEVs (compared to N‐sEVs), were defined as cancer‐specific sEV signature lost in O‐sEVs. Then, the lost portions intersected in at least four samples were obtained for the subsequent analysis. (B) Heatmap of the relative expression levels of the 169 lost mRNAs in sEVs derived from various sources. (C) Gene ontology and KEGG enrichment analyses of cancer‐specific sEV mRNAs lost in organoid culture. (D) Heatmap of the relative expression levels of the 25 lost microRNAs in sEVs derived from various sources. (E) Heatmap of KEGG pathways regulated by target genes of 4 microRNAs. (F) Venn diagram showing overlapping cancer‐specific mRNAs (*n* = 93) and miR‐190b‐5p that were lost in in vitro models but expressed in all four samples of cancer‐associated fibroblasts (CAFs)‐derived sEVs. Gene ontology biological process (G) and KEGG analyses (H) were performed for these 93 genes.

Considering the heterogeneous patterns of mRNA/microRNA expression in sEVs among individuals, data from seven completely paired samples (N‐sEVs, T‐sEVs, and O‐sEVs) were used to explore the lost genes of sEVs from in vitro models at the individual level (Figure 4A).

By taking the intersection of more than four pairs, we obtained 169 cancer‐specific sEV mRNAs lost in O‐sEVs (Figure 4B). GO and KEGG analyses showed that mRNAs related to ECM‐ and adhesion‐related pathways and immunity regulation pathways, including differentiation of mononuclear, myeloid, B and Th 1/2 cells and the regulation of T‐cell cytotoxicity, tended to be lost in the O‐sEV group (Figure 4C). Furthermore, 25 cancer‐specific sEV microRNAs were lost in O‐sEVs, with the known microRNAs including miR‐1275, miR‐1291, miR‐190b‐5p, miR‐3178, and miR‐375‐3p (Figure 4D). KEGG enrichment analysis revealed that their target genes involved many well‐known cancer‐related pathways, such as endocrine resistance, adhesion, Rap1 and MAPK signaling pathways, while there were too few target genes of miR‐375‐3p for enrichment analysis (Figure 4E).

Finally, proteomic profiling data from different samples was also analyzed. We found 37 proteins upregulated in O‐sEVs compared with N‐sEVs (Figure S3A). Surprisingly, there was no intersection with the 467 previously defined cancer‐specific sEV proteins, suggesting that the protein cargo of sEVs was much more complex in the actual TME than in vitro models. We then performed GO enrichment analysis for proteins upregulated in O‐sEVs. The results showed that most upregulated proteins were enriched in the BPs of exosome biogenesis, assembly and secretion; however, no immune‐related pathways were more enriched in O‐sEVs (Figure S3B), which was consistent with the mRNA results. Overall, we provided, for the first time, a comprehensive multiomics map of the lost sEV patterns in 3D culture models versus primary cancer tissues. Many ECM‐ and immunity regulation‐associated molecules were lost in in vitro‐derived sEVs.

### 2.5 CAF‐derived sEVs account for the majority of the lost signatures in in vitro models

Because our results strongly suggested the importance of CAFs in the tissue sEV network, we isolated fibroblasts from four BC tissues, and sEVs were extracted from the culture medium for RNA sequencing. We found that more than half of the lost sEV mRNAs in in vitro cultures were expressed in pure CAF‐derived sEVs (93 of 169) (Figure 4F). GO and KEGG analyses indicated their enrichment in ECM‐ and immune‐related regulatory pathways, including regulation of T cell‐mediated cytotoxicity and macrophage differentiation (Figure 4G‐H). In addition, miR‐190b was expressed in all CAF‐derived samples (Figure 4F), which has been reported to be the most strongly overexpressed microRNA in ER+ BC compared to the other molecular subtypes of BC or normal breast tissue.^37^

### 2.6 The distinct function of T‐sEVs and O‐sEVs in modulating tumor cells and immune cells

**FIGURE 5:**
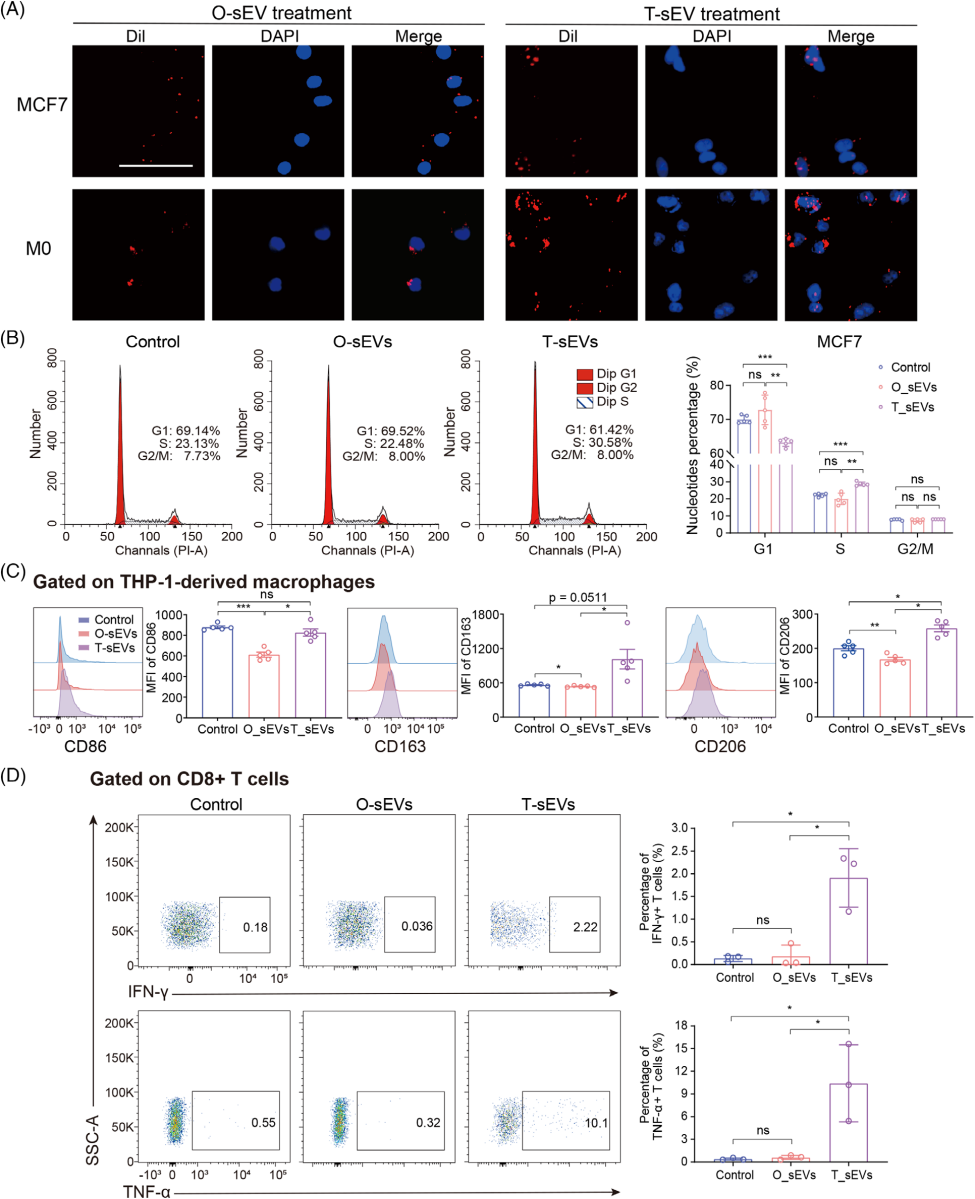
Distinct protumor and immunoregulatory capabilities of small extracellular vesicles (sEVs) derived directly from tumor tissues and in vitro organoid models. (A) Uptake of Dil‐labeled O‐sEVs (left) and T‐sEVs (right) by MCF7 and THP‐1‐derived macrophages (M0) by fluorescence microscopy. Scale bars, 50 μm. (B) Representative flow cytometric plots (left) and statistical analysis (right) of the cell cycle performed with propidium iodide DNA staining on MCF7 cells after incubation with equal amounts of O‐sEVs, T‐sEVs, or PBS for 48 h (*n* = 5). (C) Representative histograms (left) and quantification (right) for the expression levels of CD86, CD163, and CD206 on THP‐1‐derived macrophages after incubation with equal amounts of O‐sEVs, T‐sEVs, or PBS for 48 h (*n* = 5). (D) Representative contour plots (left) and quantification (right) of the expression of IFN‐γ and TNF‐α in peripheral CD8+ T cells after co‐culture with equal amounts of O‐sEVs, T‐sEVs, or PBS for 48 h, as examined by flow cytometry (*n* = 3). The above graphs show mean ± SD and paired t test. *p < 0.05, **p < 0.01, ***p < 0.001. ns, not significant.

Tumor organoids have been widely used for drug sensitivity tests, including immunotherapy, co‐cultured with immune checkpoint inhibitors and cytotoxic immune cells.^51^, ^52^, ^53^, ^54^ Because of the significant heterogeneity of the sEV cargo derived from tumor tissues and in vitro models, which indicated different biological functions, we then compared the regulatory ability of sEVs from different sources on tumor cells and immune cells. First, we directly co‐cultured BC cell lines (MCF7 and T47D) and THP‐1‐derived macrophages (M0) with T‐sEVs and paired O‐sEVs. sEVs from the two sources were both well taken up by cancer cells and macrophages (Figure 5A, Figure S4A). After 48 h of co‐culture, cells were collected for RNA sequencing. Principal component analysis (PCA) and heatmaps revealed that the control group and the T‐sEV‐ and O‐sEV‐treated groups were well separated by RNA expression profile (Figure S5). Afterward, we obtained the DEGs upregulated in the T‐sEV‐treated group (Figure S4B). GO pathway analysis revealed that the top 10 enriched BP pathways in both cancer cell lines comprised the cell cycle, cell division and DNA replication. Moreover, the upregulated genes were also enriched in pathways related to reactive oxygen species, nitric oxide, and cytokine production,^55^, ^56^ which suggested a stronger ability of T‐sEVs to activate macrophages.

We then carried out phenotypic and functional assays for validation. As expected, T‐sEVs significantly decreased the number of tumor cells in G1 phase and increased the S phase population, while the cell cycle distribution of tumor cells treated with O‐sEVs was unchanged (Figure 5B). By co‐culturing THP‐1 cells with various sEVs, T‐sEV treatment greatly induced the expression of CD86, CD163, and CD206 on M0 cells compared with O‐sEV treatment, displaying a higher level of activation and differentiation in macrophages (Figure 5C). We then conducted similar phenotypic experiments using sEVs derived from CAFs and normal fibroblasts (NF). The results revealed that CAF‐sEVs had a greater ability to increase the proportion of MCF7 cells in S phase and G2/M phase compared to NF‐sEVs and phosphate buffered saline (PBS). Additionally, CAF‐sEVs significantly induced CD86 expression on M0 cells compared to NF‐sEVs (Figure S6). Furthermore, we evaluated if the T‐sEV could activate CD8+ T cells more effectively. By co‐culturing, we found that T‐sEVs but not O‐sEVs significantly promoted the generation of interferon‐gamma(IFN‐γ) and tumor necrosis factor‐alpha (TNF‐α) in CD8+ T cells (Figure 5D). This result agreed with our previous results: mRNAs involved in regulating T cell‐mediated cytotoxicity [such as human leukocyte antigen‐DR alpha (HLA‐DRA), HLA‐B and HLA‐A] were lost in O‐sEVs (Figure 4C).

In conclusion, compared with sEVs derived from in vitro models, we proved a stronger immunostimulatory function of sEVs isolated from tumor tissues. We also implied that sEV‐related studies, especially those originating from tumor cells alone, may vastly underrepresent the biological function of the sEV network in the TME, thus the efficacy of immunotherapy evaluated in in vitro patient‐derived organoids may be underestimated.

### 2.7 Identification of the diagnostic value of cancer‐specific sEVs in BC

**FIGURE 6:**
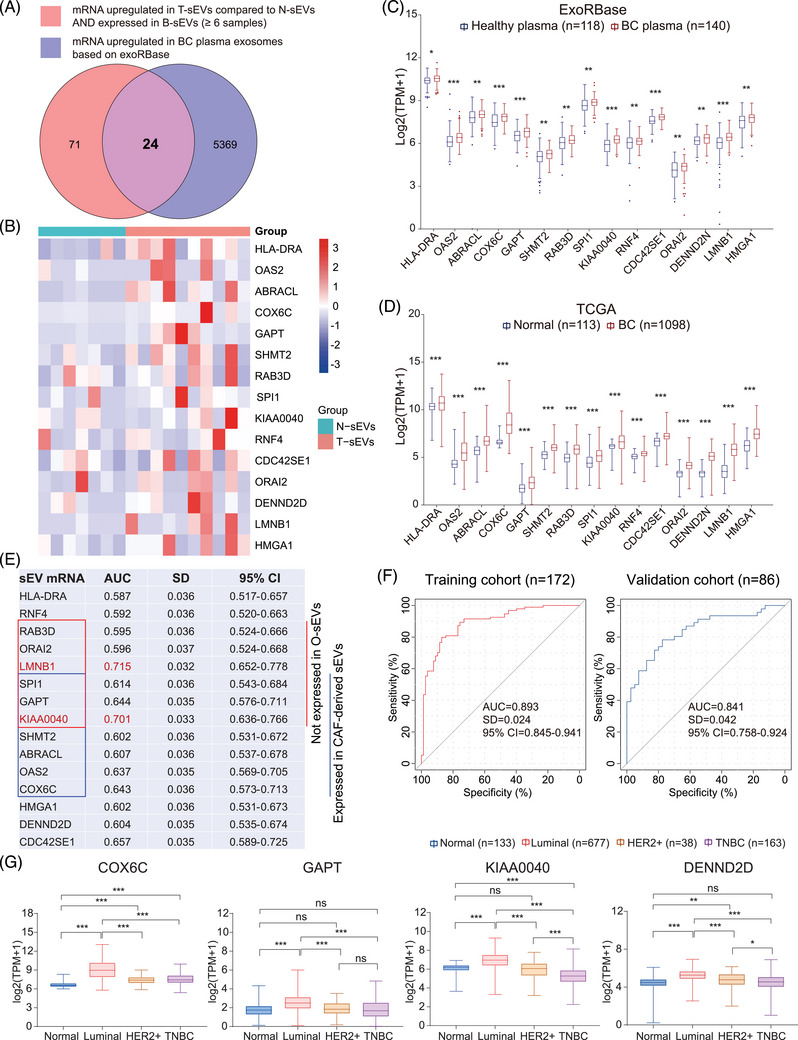
Identification of potential circulating small extracellular vesicle (sEV) biomarkers for breast cancer (BC) diagnosis. (A) 24 sEV mRNAs were identified through the intersection of cancer‐specific sEV mRNAs that were also detected in B‐sEVs (at least six samples in a total of seven samples) (*n* = 95), and mRNAs upregulated in plasma exosomes from BC patients compared with healthy people according to the exoRBase (*p* < 0.05) (*n* = 5393). (B) Heatmap of the expression levels of 15 candidate mRNAs in N‐sEVs and T‐sEVs. (C) The levels of 15 mRNAs in plasma exosomes from healthy and BC patients based on the exoRbase, and in normal and BC tissues based on TCGA‐BRCA data (D). (E) Results from receiver operating characteristic (ROC) curve analysis of these sEV‐associated mRNAs based on the exoRBase. Some were not expressed in O‐sEVs (red), which means they were not detected in at least five samples in a total of nine samples, while some were expressed in all four samples of cancer‐associated fibroblasts (CAFs)‐derived sEVs (blue). (F) ROC for the performance of the signature established using SVM in the training and validation cohorts. (G) Expression levels of COX6C, GAPT, KIAA0040, and DENND2D in normal and different subtypes of BC from TCGA database. Data are presented as medians with interquartile ranges. A two‐tailed Mann–Whitney test was used. *p < 0.05, **p < 0.01, ***p < 0.001. AUC, area under the curve; CI, confidence interval; SD, standard deviation.

Increasing evidence suggests the early diagnostic and prognostic value of circulating sEV RNA in solid tumors^57^; however, the relationship between circulating sEVs and T‐sEVs has not been well explored. Herein, we filtered 95 mRNAs upregulated in more than half (4 of 7) of T‐sEVs compared to paired N‐sEVs, which were also detected in at least six samples of B‐sEVs. Meanwhile, 5393 exosomal mRNAs were upregulated in blood samples from BC patients in the exoRBase database.^58^ Then 24 mRNAs were selected by intersecting the two gene sets (Figure 6A).

Furthermore, we found that 15 mRNAs (HLA‐DRA, OAS2, ABRACL, COX6C, GAPT, SHMT2, RAB3D, SPI1, KIAA0040, RNF4, CDC42SE1, ORAI2, DENN2D, LMNB1, and HMGA1) were also highly expressed in BC tissues based on the TCGA database (Figure 6B‐D), which could be the potential circulating diagnostic biomarkers for BC. We then evaluated the diagnostic value of the above 15 mRNAs according to the exoRBase database. The receiver operating characteristic curve (ROC) analyses identified 11 mRNAs with area under the curve (AUC) >0.6, in which LMNB1 and KIAA0040 had an AUC value > 0.7 (Figure 6E). It is worth noting that six of the mRNAs were not expressed in O‐sEVs, seven mRNAs were also expressed in CAF‐derived sEVs, and three mRNAs appeared in the intersection, suggesting that these plasma sEV biomarkers could originate from noncancer cells such as CAFs. To evaluate the combined diagnostic value, we established a diagnostic model and generated a signature for BC using the support vector machine (SVM) algorithm. The signature distinguished the BC patients from the healthy with an AUC of 0.893 (95% confidence interval [CI] = 0.845–0.941) in the training cohort. When applied to the validation cohort, the AUC of this signature was 0.841 (95% CI = 0.758–0.924) (Figure 6F). In addition, we found that the expression levels of COX6C, GAPT, KIAA0040, and DENND2D were higher in the luminal subtype than in any other molecular subtype, suggesting their specificity for luminal subtype BC (Figure 6G).

## 3 DISCUSSION

**FIGURE 7:**
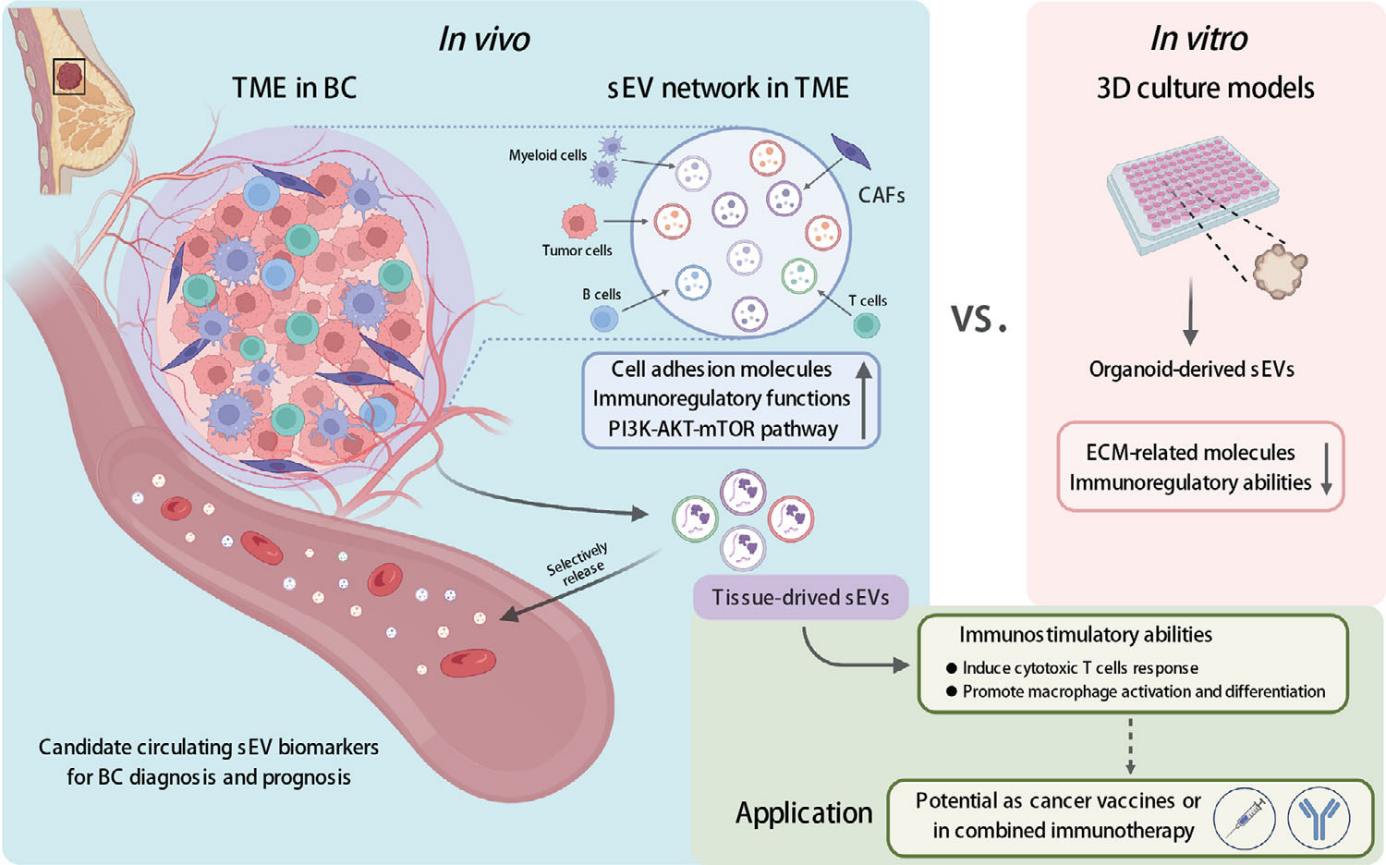
Schematic overview of the main findings in this study. Breast cancer (BC)‐specific small extracellular vesicle (sEV) network was characterized by enriched cell adhesion and immunomodulatory molecules. In vitro organoid culture model‐derived sEVs failed to entirely inherit ECM‐ and immunity regulation‐related features. Cancer‐associated fibroblasts (CAFs) contributed a large part in the sEV network. BC tissue‐derived sEVs were highlighted to present a stronger immunostimulatory function, with the potential as cancer vaccines or combined immunotherapy. Moreover, candidate sEV biomarkers in circulation with the diagnostic value could also be derived from cancer‐related stromal cells.

In recent years, mounting research based on in vitro experiments or model animals has revealed the importance of sEVs in cancer progression or immune regulation.^4^, ^21^ Still, the integrated signature of the cancer‐specific sEV network in the TME has been seldom reported. Here, we described the actual BC‐specific sEV signature in multiomics dimensions for the first time and identified the significant contribution of nontumor cells within the tissue sEV network (Figure 7).

With the deepening of sEV‐related studies, there is a growing concern regarding the drawbacks of sEVs derived from in vitro models or body fluid.^9^ For instance, cell lines failed to mimic sEVs in vivo due to long‐term cultivation and loss of interaction with other cells within the TME.^15^, ^59^, ^60^ Diverse stress conditions within TME, such as irradiation, chemotherapy, hypoxia, acidity, and starvation, have all been reported to influence EV production and contents,^61^, ^62^ as well as the uptake by recipient cells.^11^ The consequence of these differences, especially how sEVs work in actual TME is still unknown. Previous studies based on 2D cell culture showed contradictory results regarding the effect of tumor cell‐derived EVs on macrophage polarization (M1/M2).^63^, ^64^, ^65^ In our experiments here, T‐sEVs were proved to have a stronger immunostimulatory capability than O‐sEVs, indicating the immune modulatory function of sEVs derived from stromal cells may stronger than those from tumor cells. Although tumor cell‐derived EVs were reported to contribute to CD8+ T‐cell dysfunction,^66^ this phenomenon was not observed in the O‐sEV‐treated group here. Currently, numerous studies primarily focus to explore the contribution of independent cell group‐derived sEVs in tumor progression, such as cancer cells, fibroblasts, or specific immune cell populations. However, sEVs function as a highly intricate network within the TME and the previous studies were largely uncomprehensive to present the whole landscape. Our research has provided compelling evidence to demonstrate significant disparities between tumor cell‐derived sEVs (O‐sEVs) and those present in the real TME. In recent years, researchers have employed in vitro organoid models to evaluate drug sensitivity towards immunotherapy,^51^, ^54^ which turned out that EVs are greatly involved.^67^ In this context, we found an intriguing discovery that in vitro model‐derived sEVs displayed partial loss of ECM‐ and immunoregulation‐related characteristics that were observed in the real TME, underscoring that organoid‐like models may yield inaccurate results. Hence, it is crucial to develop in vitro conditions that more closely mimic the real TME in future EV‐related studies.

Considering the complexity of the EV network, determining the donor cells of specific EV contents remains a considerable challenge. Even for tumor cells, Ruivo et al. found that the protein cargoes and protumor effect of EVs from the stem and non‐stem malignant cells were highly inconsistent.^68^ EVs could inherit cell‐specific markers during the biogenesis process.^69^, ^70^ Several bioinformatic strategies combining EV sequencing data with cell‐specific genes were developed to decipher their parental cells, which have been utilized in the source tracking of urine sEVs and lung tissue‐derived EVs.^50^, ^71^ Via the similar bioinformatic strategy, our result suggested that CAFs were the cells with the most substantial ability to release sEVs by combining single‐cell transcriptomics with multiomics sEV profiles. Besides, in contrast to T‐sEVs, the majority of lost mRNAs in O‐sEVs were found in pure CAF‐derived sEVs. Abundant studies have revealed the involvement of CAF‐derived sEVs in tumor formation, progression, and immune response by in vitro co‐culture experiments.^72^ We highlighted the critical position of CAFs in the sEV network within TME for the first time. Additionally, it would be intriguing to explore the differences in the ability and contents of EVs based on the heterogeneity of CAFs.^73^ In addition, some emerging technologies have brought novel insights into this field, including characterizing single EVs with the 10x Genomics platform by clustering both EVs and their parental cells, which enabled researchers to identify specific cell origins of various EVs.^74^

The diagnostic value of tissue‐derived EVs has been reported by Jang et al., who found that EV mitochondrial membrane proteins enriched in melanoma tissue‐derived EVs were detected at high level in the plasma of cancer patients.^75^ Since our results suggested the abundance of nonmalignant cell‐derived sEVs within the tumor, we speculated that the circulating sEV‐related biomarkers could be derived from stromal components. Here, we identified circulating sEV mRNAs with diagnostic values, and a substantial proportion could be detected in pure CAF‐derived sEVs. The diagnostic efficiency of our signature is expected to be significantly higher than traditional tumor markers such as CA15‐3 and CA27‐29.^76^ Furthermore, several studies have proved the function of tumor‐related EVs in educating premetastatic niche.^77^, ^78^, ^79^ Considering the abundance of CAF‐derived sEVs in TME and their ability to release into circulation, it is rational to investigate the strategy of targeting CAFs, or specifically inhibiting the sEV production by CAFs for cancer treatment.

In addition to excavating cancer‐specific biomarkers through tumor tissue‐derived sEVs, increasing evidence also raises the great potential of tumor‐derived sEVs in immunotherapy. Several studies have demonstrated that tumor cell‐derived sEVs could efficiently deliver tumor antigens to dendritic cells, which induced specific T‐cell antitumor effects.^16^, ^29^ Meanwhile, Rao et al. also proved hepatocellular carcinoma cell‐derived sEVs were stronger than cell lysates to elicit dendritic cell‐mediated antitumor immunity.^29^ Since tumor tissue‐derived sEVs comprised tumor cell‐derived and other cancer‐associated stromal cell‐derived antigens, they may exert a superior function in stimulating antitumor immunity. Early‐stage clinical trials have explored the therapeutic strategy of using sEVs isolated from malignant ascites in late‐stage cancer patients with melanoma and colorectal cancer. Although the treatment efficiency was moderate, the ascites derived‐sEVs showed significant expression of the tumor‐specific antigens and strongly induced tumor‐specific antitumor cytotoxic T cells response,^80^, ^81^ which was in consistent with our results, that compared with O‐sEVs, T‐sEVs enriched cargoes promoting cytotoxic immunity and enhancing the IFN‐γ and TNF‐α production by CD8+ T cells. Recently, Park et al. reported a safe and efficient immunization strategy with melanoma tissue‐derived sEVs plus synthetic bacterial vesicles (SyBV) for stimulating tumor‐specific immunity featured by Th1 humoral and cellular immune responses.^82^ Furthermore, immunization with tissue‐derived sEVs plus SyBV significantly enhanced the therapeutic effect of anti‐PD‐1 monotherapy, indicating that tissue‐derived sEVs combined with immunoadjuvants could exert a synergistic effect with immune checkpoint therapy in solid tumors.

There still exist some limitations in this study. Firstly, our focus was on investigating the overall functionality of sEVs derived from various cell populations, the biological function of specific mRNA or protein was not discussed in this study. Secondly, it is of note that the majority of our tumor samples pertained to the luminal subtype. Therefore, it necessitates further explorations into cancer‐specific sEV features of TNBC or HER2‐positive BC, as this may provide valuable insights into the differential tendencies towards metastatic organs among different BC subtypes. Lastly, it is essential to validate the specific cellular origin of sEVs via alternative approaches, such as employing single‐EV sequencing technology or conducting in vitro sEV analysis after enriching specific cell types. Nonetheless, besides the transcriptomic analysis of sEVs, we successfully addressed the constraint of limited quantities of tissue‐derived sEVs by employing advanced proteomic methods. Also, we meticulously controlled the amount of peptides injected into the MS to ensure that any inter‐group differences observed were primarily attributed to variations in peptide abundance within the samples.

In conclusion, our study takes an extraordinary step forward in unraveling the intricate nature of sEVs within the TME. Given the integral role of CAFs in the sEV network, it is imperative for future investigations to delve into the immunoregulatory functions of CAF‐sEVs and their role in educating distant pre‐metastatic niches. Furthermore, by comprehensively characterizing tumor tissue‐derived sEVs, it becomes more plausible to accurately identify tumor‐specific biomarkers for liquid biopsies. This area of inquiry warrants further exploration and elucidation through large‐scale clinical trials in the future.

## 4 MATERIALS AND METHODS

### 4.1 Collection of patient specimens

Fresh BC tissues, paired normal breast tissues and peripheral blood were obtained from ten patients with BC who underwent surgical resection at the Second Affiliated Hospital of Zhejiang University School of Medicine, China. The collection of all samples was accessible with patient informed consent and approval from the Human Research Ethics Committee of the Second Affiliated Hospital of Zhejiang University School of Medicine. Tissue specimens for sEV isolation were stored with dry ice and then delivered to the laboratory immediately, while tissue specimens for the organoid models were preserved in RPMI 1640 and then delivered instantly to the laboratory at 4°C. Tissue dissociation, organoid culture, and fibroblast culture are provided in the Supplementary Materials.

### 4.2 Cell lines and cell culture

The human BC cell lines MCF7 and T47D and the human monocytic cell line THP‐1 were purchased from American Type Culture Collection (ATCC) and cultured according to the ATCC recommended culture conditions in a humidified incubator with 5% CO_2_ at 37°C. CAFs were isolated from four BC specimens by primary culture. Then, established CAFs were grown in EV‐free medium, which was collected after 48 h for sEV isolation.

### 4.3 Isolation and purification of sEVs from tissues, organoids, fibroblasts, and plasma

UC and SEC were combined to isolate sEVs from tissues, which has been proven to increase the purity and yield of sEVs.^83^ In brief, the collected tissues were first cut into 300‐μm thick slices using a Leica CM1860 freezing microtome and then incubated for 10−15 min at 37°C in 1640 medium containing Papain Dissociation System (Worthington Biochemical) according to the manufacturer's instructions. Then, the tissue digestion mixture was filtered with a 70‐μm filter and transferred into a fresh centrifuge tube containing protease and phosphatase inhibitors. After centrifugation at 300 × g for 10 min and 2000 × g for 10 min at 4°C to eliminate cells and tissue debris, the supernatant was further centrifuged at 10000 × g for 20 min at 4°C and then processed through a 0.22 μm filter (Millipore, USA). The supernatant was ultracentrifuged at 150000 × g for 2 h at 4°C, and the pellet was then resuspended in 1‐mL PBS, followed by purification of sEVs using Exosupur columns (Echobiotech, China) in accordance with the manufacturer's instructions and ultrafiltration.

The sEVs of tumor organoid and fibroblast culture medium were isolated by the SEC method. First, the collected culture medium was centrifuged at 300 × g for 10 min and 2000 × g for 10 min at 4°C to remove cell debris. The resulting supernatant was filtered using a 0.22‐μm filter and further concentrated to 2‐mL using ultrafiltration tubes (100 kDa cutoff, Millipore). The concentrated medium was then loaded into Exosupur columns, which were washed with a 3× bed volume of PBS ahead. Subsequently, 1‐mL PBS was added to the column for each fraction, and sEVs were enriched in fractions 5−9 for collection.

Whole blood from patients was first centrifuged twice at 2500 × g for 15 min at 4°C to eliminate cellular components. The plasma was transferred to a new tube, then ultrafiltration and SEC were applied for the isolation of sEVs from plasma as described above. The experimental procedures to obtain sEVs from all kinds of samples are displayed in detail in Figure S7.

### 4.4 Characteristics of sEVs

#### 4.4.1 NTA

The concentration of purified sEVs was first diluted to the optimal range of detection (1 × 10^7^/mL and 1 × 10^9^/mL). The size and quantity of sEVs were obtained using a ZetaView PMX 110 (Particle Metrix, Meerbusch, Germany) equipped with a 405 nm laser. For each sample, a video of 60‐sec duration was taken with a frame rate of 30 frames/second, and NTA software was used to analyze the partial movement.

#### 4.4.2 TEM

The morphology of purified sEVs was detected by TEM analysis. Briefly, 10‐μl sEV solution was applied to a copper mesh for 1 min at room temperature and washed with sterile distilled water. Then, the sEVs were stained with 2% uranyl acetate for 1 min, and all samples were further observed and photographed under a TEM (H‐7650, Hitachi Ltd., Tokyo, Japan).

#### 4.4.3 Western blot analysis

The protein concentration of sEVs and the whole cell lysis were detected by a bicinchoninic acid (BCA) protein quantitation kit (Beyotime, China). Each sample was denatured in 5× sodium dodecyl sulfonate (SDS) buffer at 95°C for 10 min. Subsequently, proteins were separated by 4%−12% (gradient) SDS‒PAGE and then transferred to PVDF membranes. After blocking with 5% nonfat dry milk for 1 h at room temperature, the membranes were incubated with the corresponding primary antibodies Alix (ab186429, Abcam), Tsg101 (ab125011, Abcam), HSP70 (ab181606, Abcam), CD9 (A1703, Abclonal), CD63 (sc‐5275, Santa), and Calnexin (10427‐2, Proteintech) overnight at 4°C. After incubation with the secondary antibody for 1 h at room temperature, the blots on the membranes were visualized using a Tanon4600 Automatic chemiluminescence image analysis system (Tanon, Shanghai, China).

### 4.5 sEV RNA sequencing and analysis

sEV RNA isolation and detection, library preparation and sequencing, and RNA‐seq analysis are provided in the Supplementary Materials.

### 4.6 sEV protein extraction and analysis

Pressure cycling technology (PCT)‐based lysis and peptide extraction, and LC‐MS/MS analysis are provided in the Supplementary Materials.

### 4.7 ScMappR

scMappR^47^ was used to calculate cwFold change by combining DEGs, a cell‐type expression matrix, and bulk sEV mRNA expression. For each DEG, the cwFold change was sorted by cell type to discover which cell types are most likely responsible for the differential expression. The cell‐type expression matrix was obtained from a published study that analyzed 26 primary pretreatment breast tumors through single‐cell RNA sequencing.^48^

### 4.8 Human CD8+ T‐cell enrichment

First, human peripheral blood mononuclear cells (PBMCs) were isolated by density centrifugation (800 g, 15 min) from peripheral blood with a human lymphocyte separating fluid (Dakewe Biotech). Then, naïve CD8+ T cells were enriched using CD8 MicroBeads (Miltenyi Biotec) to >90% purity according to the manufacturer's instructions.

### 4.9 sEV uptake and co‐culture studies

sEVs isolated from tissues and organoids were collected and labeled with the fluorescent lipophilic dye DiIC_18_(3) (Yeasen Biotech, Shanghai, China). Briefly, a total of 20 μg sEVs were resuspended in 1‐mL PBS containing 1‐μL DiI following incubation at 37°C for 30 min. After being washed with PBS and ultracentrifuged, labeled sEV pellets were resuspended and added to the macrophages or BC cells. After 24 h of sEV treatment, cells for fluorescence microscopy underwent fixation, permeabilization and nuclear staining (DAPI; Abcam, MA, USA). sEV uptake images were captured on a Leica DM IL microscope (Leica Microsystems, Wetzlar, Germany). For co‐culture experiments, macrophages, and BC cells were seeded in 24‐well cell culture plates and treated with 20 μg of the indicated sEVs or an equal volume of PBS. Cells were harvested after 48 h for RNA sequencing. For another phenotypic experiment, macrophages, CD8+ T cells and BC cells were seeded in 48‐well cell culture plates and treated with 10 μg of the indicated sEVs or an equal volume of PBS. Corresponding cells and supernatant were collected after 48 h for flow cytometry.

### 4.10 Flow cytometry

To analyze cell surface marker expression, cells were suspended in a solution of 2% fetal bovine serum (FBS) in PBS and incubated with fluorescence‐labeled antibodies specific to the respective markers at 4°C for 30 min. The following antibodies were utilized for flow cytometry: PE anti‐CD86, FITC anti‐CD163 and APC anti‐CD206 (BioLegend). FVS510 staining was used to exclude dead cells. To perform intracellular staining analysis, CD8+ T cells underwent fixation and permeabilization using a BD Cytofix/Cytoperm Soln Kit following surface staining, and then intracellular APC anti‐IFN‐γ and PE/Cy7 anti‐TNF‐α staining were performed to detect the cytokine production of CD8+ T‐cell populations.

### 4.11 Cell cycle analysis

BC cells were treated with sEVs from different sources for 48 h and harvested to detect the cell cycle by flow cytometric analysis using a Cell Cycle Staining Kit (LiankeBio, Hangzhou, China) according to the manufacturer's instructions. Briefly, cells were resuspended in 500 μL of cell cycle staining buffer with the addition of 5 μL of permeabilization solution. Then, the solution was mixed by vortexing for 10 s and incubated for 30 min at room temperature in the dark. The cell cycle distribution was analyzed using ModFit 5 software after measuring the DNA content of samples by a CytoFLEX Flow Cytometer (Beckman Coulter).

### 4.12 Immunohistochemistry of tumor tissue and organoids

Tumor slices and organoids were fixed in 4% paraformaldehyde overnight followed by dehydration, paraffin embedding, sectioning, and standard H&E staining as previously described.^84^ For cryosections, samples were fixed in 4% paraformaldehyde in PBS overnight at 4°C, followed by immersion in 30% sucrose solution in PBS and subsequent embedding in optimal cutting temperature compound (OCT) medium. The primary antibodies used for immunostaining analysis were anti‐ER (Abcam, ab108398), anti‐PR (Abcam, ab16661), anti‐HER2 (CST, 4290), anti‐p‐PI3K (Affinity, AF3241), anti‐p‐AKT (Abcam, ab8933), and anti‐p‐mTOR (Abcam, ab109268) antibodies. Biotinylated or fluorescent secondary antibodies were used for detection and visualization. Nuclei were counterstained with Harris hematoxylin for immunohistochemistry or DAPI for immunofluorescence staining. Images were obtained using a Nikon SMZ1500 inverted microscope (Nikon). Confocal images were obtained with a Zeiss LSM T‐PMT confocal laser‐scanning microscope (Carl Zeiss).

### 4.13 SVM model

The expression data of blood exosomal mRNA in healthy (*n* = 118) and BC (*n* = 140) was downloaded from the exoRBase database (http://www.exorbase.org/).^58^ The sample was randomly divided into training (*n* = 172) and validation (*n* = 86) cohorts at a 7:3 ratio. The predictive model was built based on SVM by using LibSVM package.^85^ The training set was used to automatically obtain the optimal parameters for model establishment through an easy search method of internal cross‐validation. The model was then verified using the validation cohort.

### 4.14 Systematic analysis

Functional and pathway enrichment analyses were performed using the ClusterProfiler package^86^ with GO terms and KEGG pathways. Single‐sample GSEA (ssGSEA) was performed using the GSVA package^87^ with default settings. The mRNA/microRNA‐pathway network was constructed by Cytoscape software. The PPI network was constructed in the STRING database (http://string‐db.org) using curated interaction databases and experimentally established under default settings.

### 4.15 Statistical analysis

Statistical analysis was performed using Prism 9.0 analysis software. Data are expressed as the means and standard errors of the mean, or the medians and interquartile ranges. The Mann–Whitney *U* test, Student *t* test, or chi‐square test were used to compare quantitative variables between two groups. A *p*‐value < 0.05 or *q*‐value < 0.05 were considered to be statistically significant.

## AUTHOR CONTRIBUTIONS

CN, MJ, and JH designed and supervised the study. WZC, JZ, JL, SSS, WJX, XYY, JS, WLW, and JXJ collected clinical sample. ZCW and MJ performed organoid and tissue slice culture. LSS and HHH analyzed the data of transcriptomes and proteomes and performed the experiments. JXL, YY, and TZ contributed to data analysis. CN, LSS, and HHH conducted statistical analysis, prepared figures, and wrote the original draft. All authors read and approved the final manuscript.

## CONFLICT OF INTEREST STATEMENT

The authors declare no conflict of interest.

## ETHICS STATEMENT

This study was approved by the Human Research Ethics Committee of the Second Affiliated Hospital of Zhejiang University School of Medicine and was conducted according to the Declaration of Helsinki principles (approval number: 2022‐0827). Signed informed consent was obtained from each participant.

## Supporting information

Supporting Information

Supporting Information

Supporting Information

## Data Availability

The raw sequencing data of sEV transcriptomes and proteomes in this paper have been deposited at the Genome Sequence Archive at the National Genomics Data Center (Beijing, China) under the BioProject ID PRJCA013091. The mass spectrometry proteomics data have been deposited to the ProteomeXchange Consortium (http://proteomecentral.proteomexchange.org) via the iProX partner repository with the dataset identifier PXD046409. All data are available from the corresponding authors upon request.
