# Supplementary material for: Integrated transcriptomics, proteomics, and functional analysis to characterize the tissue‐specific small extracellular vesicle network of breast cancer
Source: MedComm (2020). 2023 Dec 3;4(6):e433. doi: 10.1002/mco2.433 (PMC10694390; doi:10.1002/mco2.433)
Supplement: Supplementary file 1 — Supporting Information [file MCO2-4-e433-s001.docx]

**Integrated transcriptomics, proteomics and functional analysis to characterize the tissue-specific small extracellular vesicle network of breast cancer**

**Running title: Small EVs derived from BC tissues and organoids**

**Authors:** Lesang Shen^1,2,3#^, Huanhuan Huang^1,2,3#^, Zichen Wei^4,5#^, Wuzhen Chen^1,2,3#^, Jiaxin Li^1,2,3^, Yao Yao^1,2,3^, Jun Zhou^6^, Jian Liu^6^, Shanshan Sun^1,2,3^, Wenjie Xia^7^, Ting Zhang^2,3,8^, Xiuyan Yu^1,2,3^, Jun Shen^9^, Weilan Wang^10^, Jingxin Jiang^1,2,3^, Jian Huang^1,2,3*^, Ming Jiang^4,11*^, Chao Ni^1,2,3*^

**Affiliations:**

^1^Department of Breast Surgery, Second Affiliated Hospital, Zhejiang University, Hangzhou, Zhejiang, China.

^2^Key Laboratory of Tumor Microenvironment and Immune Therapy of Zhejiang Province, Second Affiliated Hospital, Zhejiang University, Hangzhou, Zhejiang, China.

^3^Cancer Center, Zhejiang University, Hangzhou, Zhejiang, China.

^4^Center for Genetic Medicine, the Fourth Affiliated Hospital, Zhejiang University School of Medicine, Hangzhou, Zhejiang, China.

^5^Department of Anesthesiology, Taihe Hospital, Hubei University of Medicine, Shiyan, Hubei, China.

^6^Department of Breast Surgery, Affiliated Hangzhou First People’s Hospital, Zhejiang University, Hangzhou, Zhejiang, China.

^7^Department of Breast Surgery, Zhejiang Provincial People’s Hospital, Hangzhou, Zhejiang, China.

^8^Department of Radiation Oncology, Second Affiliated Hospital, Zhejiang University, Hangzhou, Zhejiang, China.

^9^Department of Surgical Oncology, Sir Run Run Shaw Hospital, Zhejiang University, Hangzhou, Zhejiang, China.

^10^Department of Breast Surgery, Changxing People's Hospital, Huzhou, Zhejiang, China.

^11^Zhejiang Provincial Key Laboratory of Genetic & Developmental Disorders, Hangzhou, Zhejiang, China.

*Corresponding authors

#These authors contributed equally to this work

**This PDF file includes:**

Materials and Methods

Figures S1 to S7

Tables S1 and S4

**Materials and Methods**

**Tissue dissociation and organoid culture**

Samples were washed three times with cold PBS with antibiotics and cut into 2-3 mm^3^ pieces. For organoid culture, tumor pieces were minced and digested in 1 mg/ml collagenase I (Worthington) and 0.001% DNase (Sigma‒Aldrich) in Advanced DMEM/F12 medium (Gibco) at 37 °C for 1-2 h with intermittent agitation. After digestion, the suspensions were repeatedly triturated by pipetting and strained over 70 µm cell strainers (BD Falcon). The suspensions were centrifuged at 400 × g for 5 min, and the pellet was resuspended in 10 ml Advanced DMEM/F12 and centrifuged again at 400 × g. In the case of a visible red pellet, erythrocytes were lysed in 1 mL red blood cell lysis buffer (Roche) for 5 min at room temperature followed by dilution with 10 ml Advanced DMEM/F12 medium and centrifugation at 400 × g. Cells were counted and resuspended in 10 mg/ml cold Cultrex growth factor reduced BME type 2 (Bio-techne). Then, 20 µL drops of BME-cell suspension were plated and allowed to solidify on prewarmed 48-well culture plates (Corning) at 37 °C for 15 min. After solidification, 250 µl of BC organoid culture medium (Advanced DMEM/F12 supplemented with 1× N2 (Invitrogen) and 1× B27 (Invitrogen), 1× GlutaMAX (Life Technologies), 1× HEPES, 1× penicillin–streptomycin (Gibco), 1 mM N-acetyl-l-cysteine (Sigma), 10 mM nicotinamide (Sigma), 50 ng/ml epidermal growth factor (Invitrogen), 100 ng/ml noggin (R&D Biosystems), 50 ng/ml Wnt3A (Biogenous), 100 ng/ml R-spondin (Sino Biological), 100 ng ml−1 Fgf10 (R&D Biosystems), 10 μM SB202190 (Sigma) and 500 nM A8301 (Tocris)) was added to each well, and the plates were transferred to humidified 37 °C 5% CO_2_ incubators as described previously^1^. The medium was changed every 4 days, and the organoids were passaged after 1-3 weeks. For passaging, the organoids were disrupted either by mechanical shearing or digestion with TrypLE Express (Gibco). The organoid fragments were resuspended in cold BME and reseeded as above at 1:3–1:6 ratios to allow the formation of new organoids. All organoid lines routinely tested negative in the MycoAlert mycoplasma detection kit (Lonza).

**Fibroblast culture**

Freshly isolated surgical samples were placed in pre-cooled DMEM containing 10% FBS. The tissue was washed 3 times with PBS and cut into ~1 mm^3^ pieces. The tissue pieces were placed in 15 ml centrifuge tubes with 5-10 ml of collagenase I, and digested for 30 min at 37°C. The tissue pieces digestion was terminated by adding an 10x volume of DMEM. The tissue homogenate was filtered through a 70 mm filter membrane, and the filtered cell suspension was centrifuged at 1000 rpm for 4 min at 4°C. The cells were resuspended using DMEM containing 10% FBS. The cells were seeded uniformly in a 10 cm dish, and placed in a 5% CO2 incubator at 37°C for 40 min. The supernatant was aspirated to remove non-fibroblasts and dead cells, and then 10 ml of DMEM containing 10% FBS was added to continue the culture.

**sEV RNA sequencing and analysis**

**sEV RNA isolation and detection**

Total RNA was extracted and purified from sEV pellets using a miRNeasy Kit (Qiagen, CA, USA) according to the manufacturer’s instructions. RNA concentration, integrity and purity were quantified using an Agilent Bioanalyzer 2100 (Agilent Technologies, CA, USA).

**Library preparation and sequencing**

The RNA-seq libraries were prepared from 250 pg-10 ng input RNA material using SMARTer Stranded Total RNA-Seq Kit V2 (Takara Bio USA, Inc.), and the small RNA libraries were prepared from 1 ng-500 ng RNA per sample using a QIAseq microRNA Library Kit (Qiagen) following the manufacturer’s recommendations. Index codes were adopted to attribute sequences to each sample. Reverse-transcription primers containing unique molecular indices (UMIs) were introduced to quantify microRNA expression during library amplification. The library quality and quantity were then assessed on an Agilent Bioanalyzer 2100 (Agilent). Cluster generation was performed on a cBot Cluster Generation System using TruSeq PE Cluster Kitv3-cBot-HS (Illumina, CA, USA) according to the manufacturer’s instructions. Then, the library preparations were sequenced on an Illumina NovaSeq 6000 platform (Illumina) and paired-end reads were generated.

**RNA-seq analysis**

Raw data (raw reads) in Fastq format were processed through in-house Perl scripts to remove reads containing adapter, poly-N and low-quality reads from the raw data. Meanwhile, the Q20, Q30, GC content and sequence duplication level of the clean data were calculated for quality control. The clean paired-end reads were mapped to the reference genome GRCh38 using TopHat2/Bowtie2 software. Only uniquely mapped reads were used for the quantification of gene expression.

For lncRNA analysis, transcript assembly was performed using StringTie and Scripture. Subsequently, the Cuffcompare program from the Cufflinks package was used to annotate the assembled transcripts, and the unknown transcripts were used to screen for putative lncRNAs. Putative protein-coding RNAs were filtered based on a minimum length and exon number threshold. Transcripts with more than 200 nt in length and with over two exons were selected as lncRNA candidates, and they were further screened using four computational approaches (CPC/CNCI/Pfam/CPAT) to distinguish nonprotein coding genes from protein-coding genes. The different types of lncRNAs were identified using Cuffcompare. In the second step, StringTie was used to calculate fragments per kilobase per million mapped reads (FPKM) of coding genes and lncRNAs in each sample.

For microRNA analysis, clean readings were aligned and compared with databases including Silva, GtRNAdb, Rfam, and Repbase databases with the help of Bowtie software. Then, ribosomal RNA (rRNA), transfer RNA (tRNA), small nuclear RNA (snRNA) and small nucleolar RNA (snoRNA) were excluded. The remaining reads were used for microRNA identification by mapping known microRNAs from the Human Genome GRCh38 and miRbase. EdgeR was used to normalize microRNA expression by transforming UMI counts to counts per million (CPM).

Differential expression analysis was performed using the EdgeR package (including group comparisons and two sample comparisons), and genes with a fold change (FC) ≥ 1.5 or ≤ -1.5 and a p-value < 0.05 were considered to be significantly differentially expressed.

**sEV protein extraction and analysis**

**Pressure cycling technology (PCT)-based lysis and peptide extraction**

Freeze-drying of the samples was first performed, followed by PCT-assisted sample lysis and protein digestion and PulseDIA-PASEF analysis, as described previously, which is suitable for minute amounts of samples^2, 3^. sEV samples were placed in PCT-MicroTubes with 30 µl lysis buffer containing 6 M urea (Sigma‒Aldrich) and 2 M thiourea (Sigma‒Aldrich), 10 µl 100 mM Tris (2-carboxyethyl) phosphine (Adamas-beta), 5 µl 800 mM iodoacetamide (Sigma‒Aldrich) and 5 µl 100 mM ammonium bicarbonate (GENERAL-REAGENT) using a barocycler (model NEP2320-45k, Thermo Fisher Scientific). Lysis was performed with 90 cycles of high pressure (45,000 psi, 30 s per cycle) and ambient pressure (10 s per cycle). The extracted sEV proteins then underwent reduction and alkylation before protein digestion. Lys-C (0.5 µg, Hualishi Tech) and 0.5 µg trypsin (Hualishi Tech)-mediated proteolysis was performed under 90 cycles of pressure alternation (20,000 psi for 50 s per cycle and at ambient pressure for 10 s per cycle). Subsequently, samples were transferred to a 1.5 ml tube, and then 15 µl 10% trifluoroacetic acid (Thermo Fisher Scientific) was added for a final concentration of 1% to stop digestion with the final pH 2-3. The digested protein samples were centrifuged at 12,000 × g for 5 min, and the supernatant was collected. Then, desalting was performed using Pierce^TM^ C18 spin column (Thermo Fisher Scientific) following the manufacturer’s instructions.

**LC‒MS/MS analysis**

Based on traditional diaPASEF technology, PulseDIA-PASEF divides a window into multiple windows with a smaller range according to the mass-to-charge ratio and evenly distributes these windows to different mass spectrometry methods in a pulsed manner for acquisition. The same sample is divided into multiple short gradient injections, each injection method has a different mass spectral window, and finally, the mass spectral data collected several times are combined and analyzed^4^.

The sample was analyzed on a nanoElute UHPLC (Bruker Daltonics, Germany) coupled to a timsTOF Pro (Bruker Daltonics, Germany) equipped with a CaptiveSpray ion source. Peptide powder was reconstituted in buffer A (0.1% formic acid in water). Peptide digests were separated on a 15 cm analytical column (75 µm ID, 1.9 µm, C18 beads, homemade) at a flow rate of 300 nL/min using a 60 min gradient with an integrated Toaster column oven at 50 °C. Mobile phase B was acetonitrile with 0.1% formic acid. The phase of B was increased from 5% to 27% in 50 min, 27% to 40% in 10 min, and 37% to 80% in 1 min and was sustained at 80% for 10 min. The timsTOF Pro was operated in positive ion data-dependent acquisition parallel accumulation serial fragmentation (PASEF) mode. The capillary voltage was set to 1400 V. The MS and MS/MS spectra were acquired from 100 to 1,700 m/z, and an ion mobility range (1/K0) from 0.7 to 1.3 vs./cm2. The accumulation and ramp time were set to 100 ms to achieve a duty cycle close to 100%. To perform diaPASEF acquisition mode, we defined two 15 Th isolation windows from m/z 384 to 1,023 and m/z from 475 to 1114 (ref. ^5^).
 Raw files and quality control samples were processed using Spectronaut (version 14.6). The final generated library contained 13,624 protein groups and 448,338 peptide precursors. A total of 8720 proteins were identified. Differential expression analysis was performed on log_2_ transformed data using the Limma package to identify significant proteins (FC > 2, p-value < 0.05).

**References**

1. Sachs N, de Ligt J, Kopper O, et al. A Living Biobank of Breast Cancer Organoids Captures Disease Heterogeneity. *Cell*. 2018;172(1-2):373-386.e10.

2. Guo T, Kouvonen P, Koh CC, et al. Rapid mass spectrometric conversion of tissue biopsy samples into permanent quantitative digital proteome maps. *Nat Med*. 2015;21(4):407-13.

3. Cai X, Xue Z, Wu C, et al. High-throughput proteomic sample preparation using pressure cycling technology. *Nat Protoc*. 2022;

4. Cai X, Ge W, Yi X, et al. PulseDIA: Data-Independent Acquisition Mass Spectrometry Using Multi-Injection Pulsed Gas-Phase Fractionation. *J Proteome Res*. 2021;20(1):279-288.

5. Meier F, Brunner AD, Frank M, et al. diaPASEF: parallel accumulation-serial fragmentation combined with data-independent acquisition. *Nat Methods*. 2020;17(12):1229-1236.


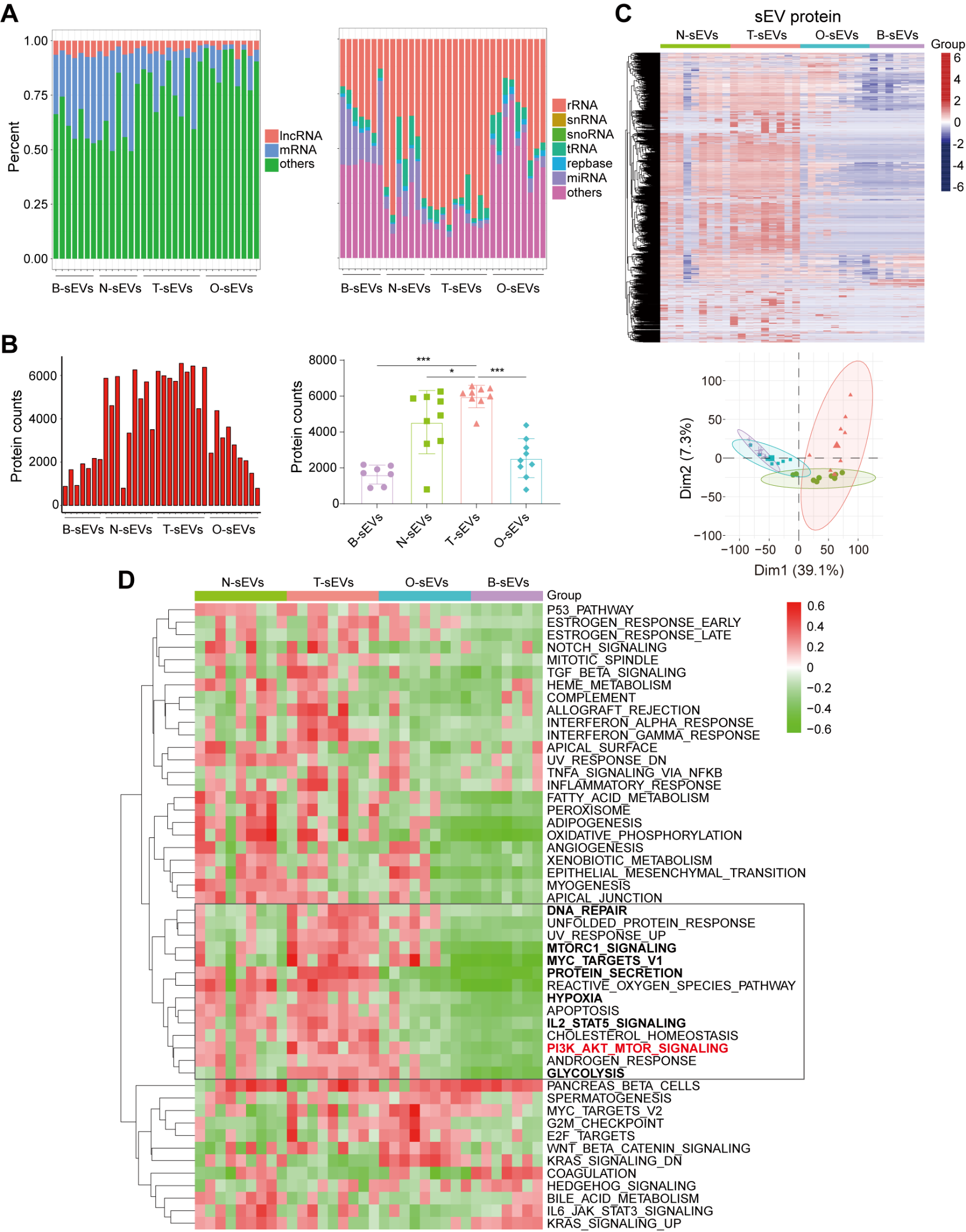


**Figure S1. The landscape of transcriptomic and proteomic profiles of sEVs from all involved samples.** (A) Frequency of different types of RNA represented in the library of each sEV sample. (B) The number of detectable proteins observed in LC-MS/MS analysis in each sEV sample (left) and group (right). Data are presented as the mean ± SD by Student’s t test. (C) Heatmap and PCA plot of detected sEV proteins in all sEV samples from different sources. Unsupervised clustering is shown. (D) Heatmap showing the degree of enrichment in hallmark notable pathways for sEVs derived from different sources. *p < 0.05, ***p < 0.001.


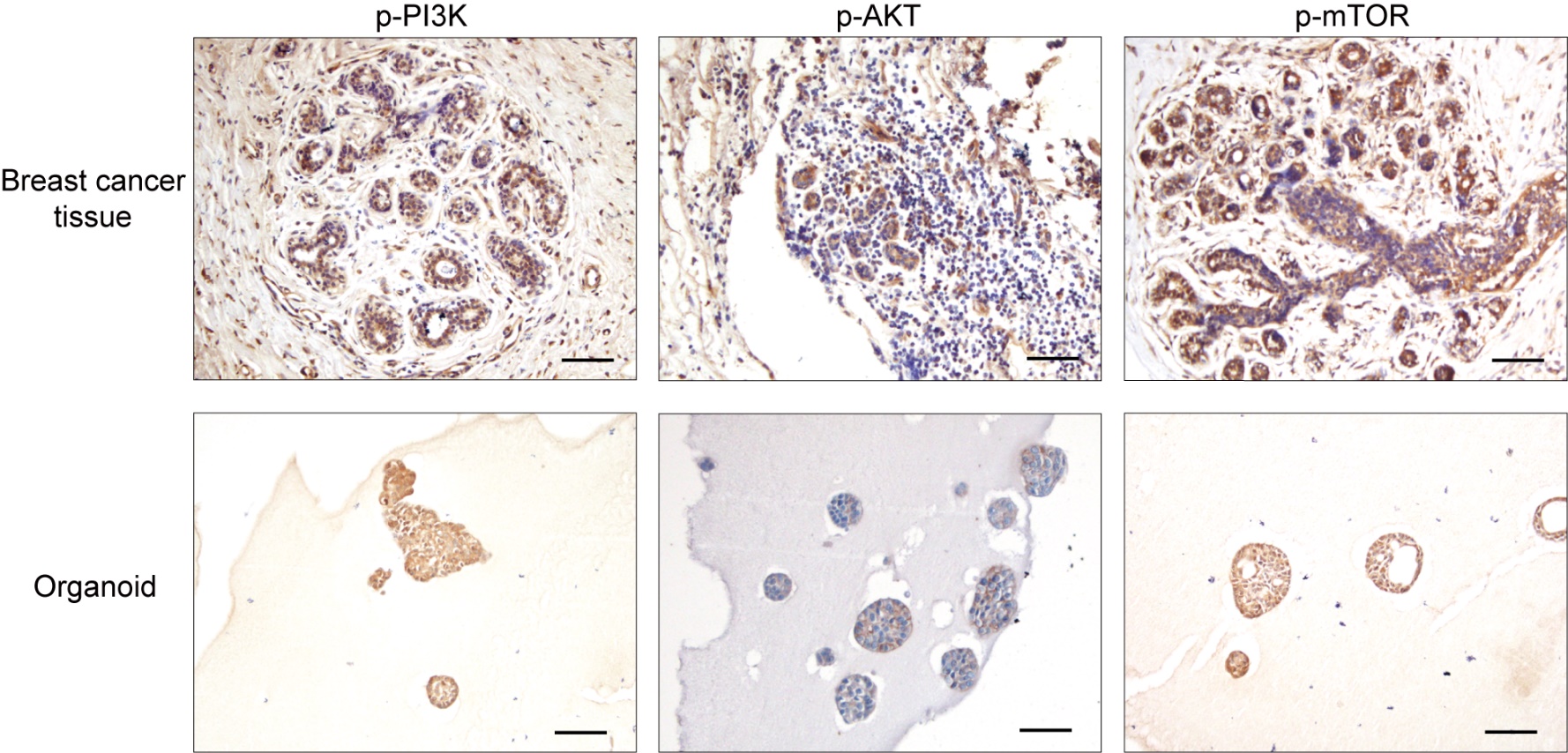


**Figure S2. The PI3K-AKT-mTOR pathway is more highly activated in BC tissue than in organoids.** Representative immunohistochemical staining for p-PI3K, p-AKT and p-mTOR expression in BC tissue and paired organoid. Scale bars, 100 µm.


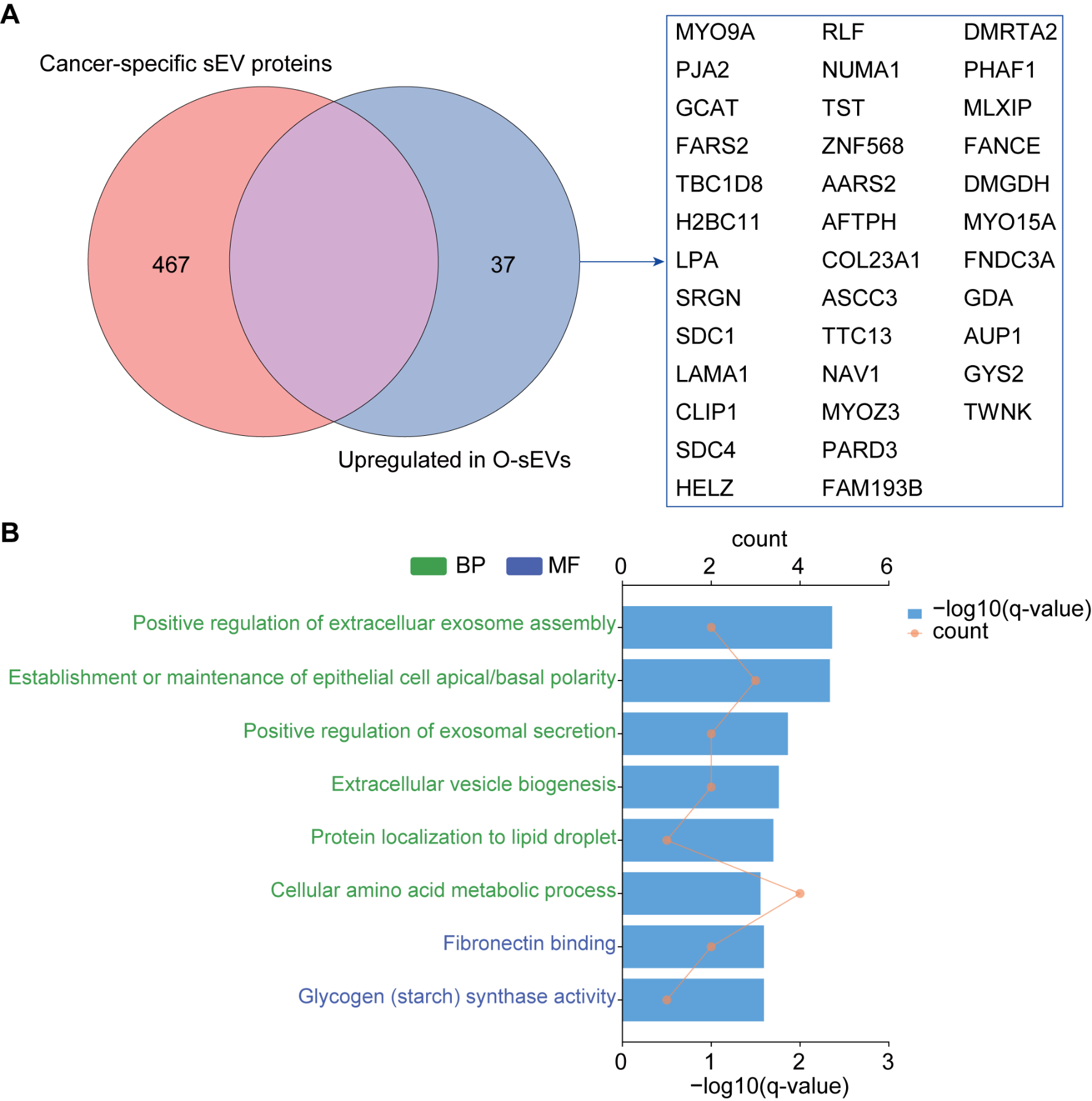
\

**Figure S3. The protein cargoes of sEVs vary widely between the actual tumor microenvironment and *in vitro* models.** (A) List of proteins upregulated in O-sEVs (n = 37) compared with N-sEVs (fold change > 2 and p < 0.05). (B) Biological process and molecular function enrichment analysis of proteins upregulated in O-sEVs.


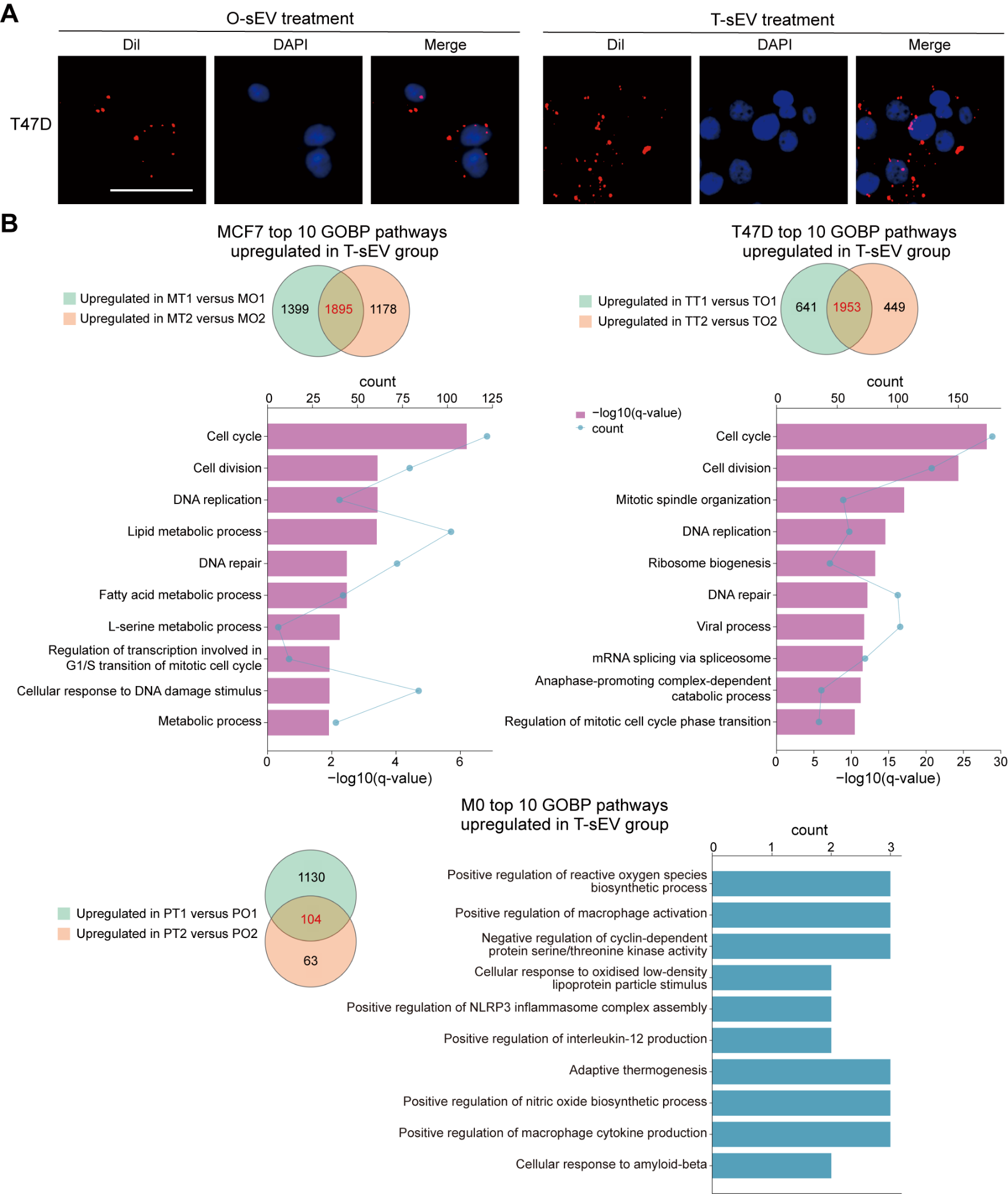


**Figure S4. Different effects of sEVs derived directly from tumor tissues and *in vitro* organoid models on the tumor cell cycle and macrophage regulation.** (A) Uptake of Dil-labeled O-sEVs (left) and T-sEVs (right) by T47D through fluorescence microscopy. Scale bars, 50 µm. (B) MCF7, T47D and M0 treated with equal amounts of O-sEVs and T-sEVs after 48 h were collected for RNA sequencing (2 paired samples with 2 biological replicates). Bar diagrams showing the top 10 enriched biological process pathways of genes upregulated in the T-sEV treatment group compared with the O-sEV treatment group for MCF7, T47D and M0. The upregulated genes were obtained by generating the intersection of the two samples. MT, MCF7 treated with T-sEVs; MO, MCF7 treated with O-sEVs; TT, T47D treated with T-sEVs; TO, T47D treated with O-sEVs; PT, M0 treated with T-sEVs; PO, M0 treated with O-sEVs.


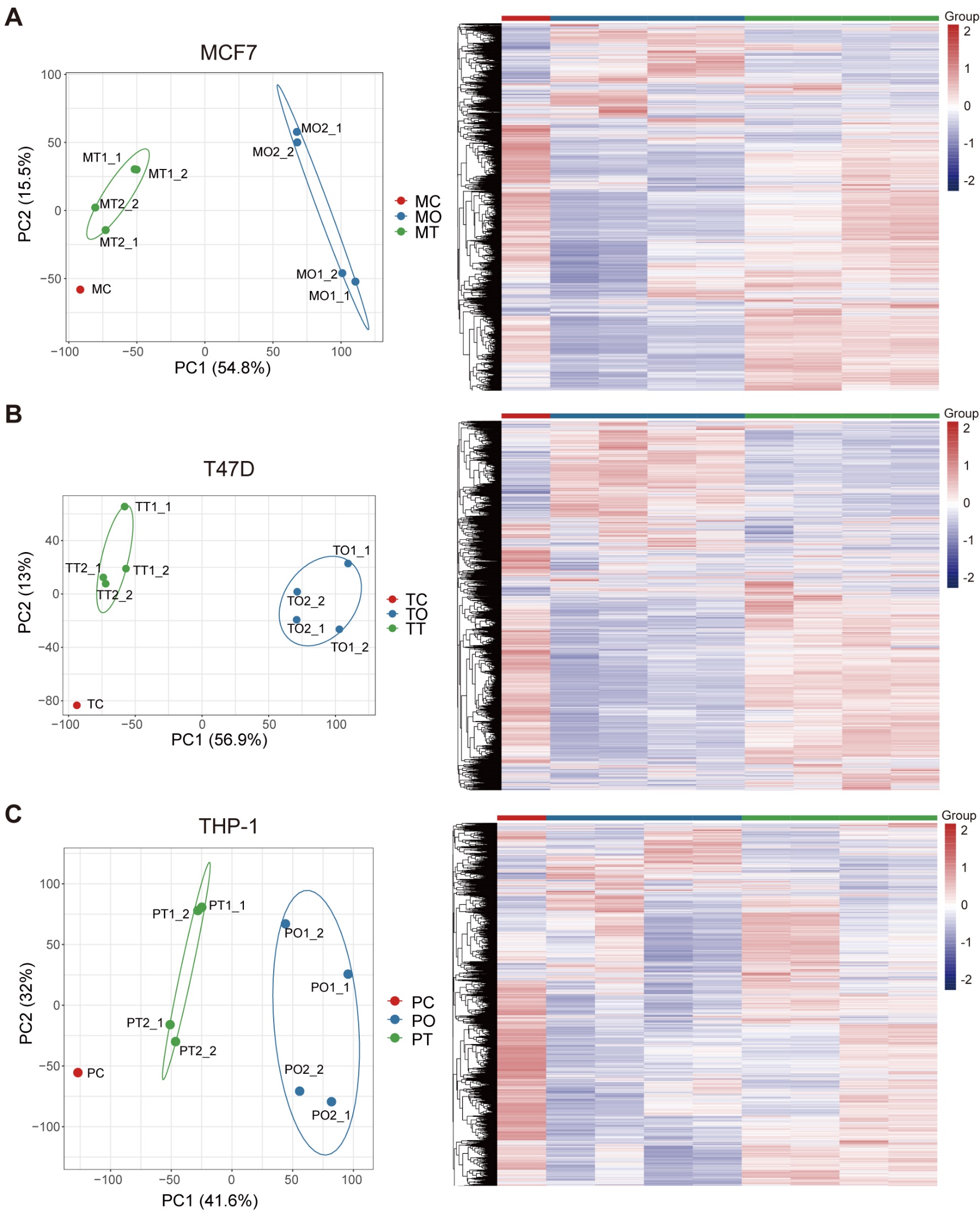


**Figure S5. BC cells and macrophages treated with sEVs derived directly from tumor tissues and *in vitro* organoid models have distinct transcriptomic signatures.** PCA plots and heatmaps of MCF7 (A) T47D (B) and THP-1-derived macrophages (C) treated with equal amounts of O-sEVs and T-sEVs, using PBS treatment as a comparator (control: n = 1; O-sEVs and T-sEVs treated groups: n = 2 with 2 biological replicates).

**
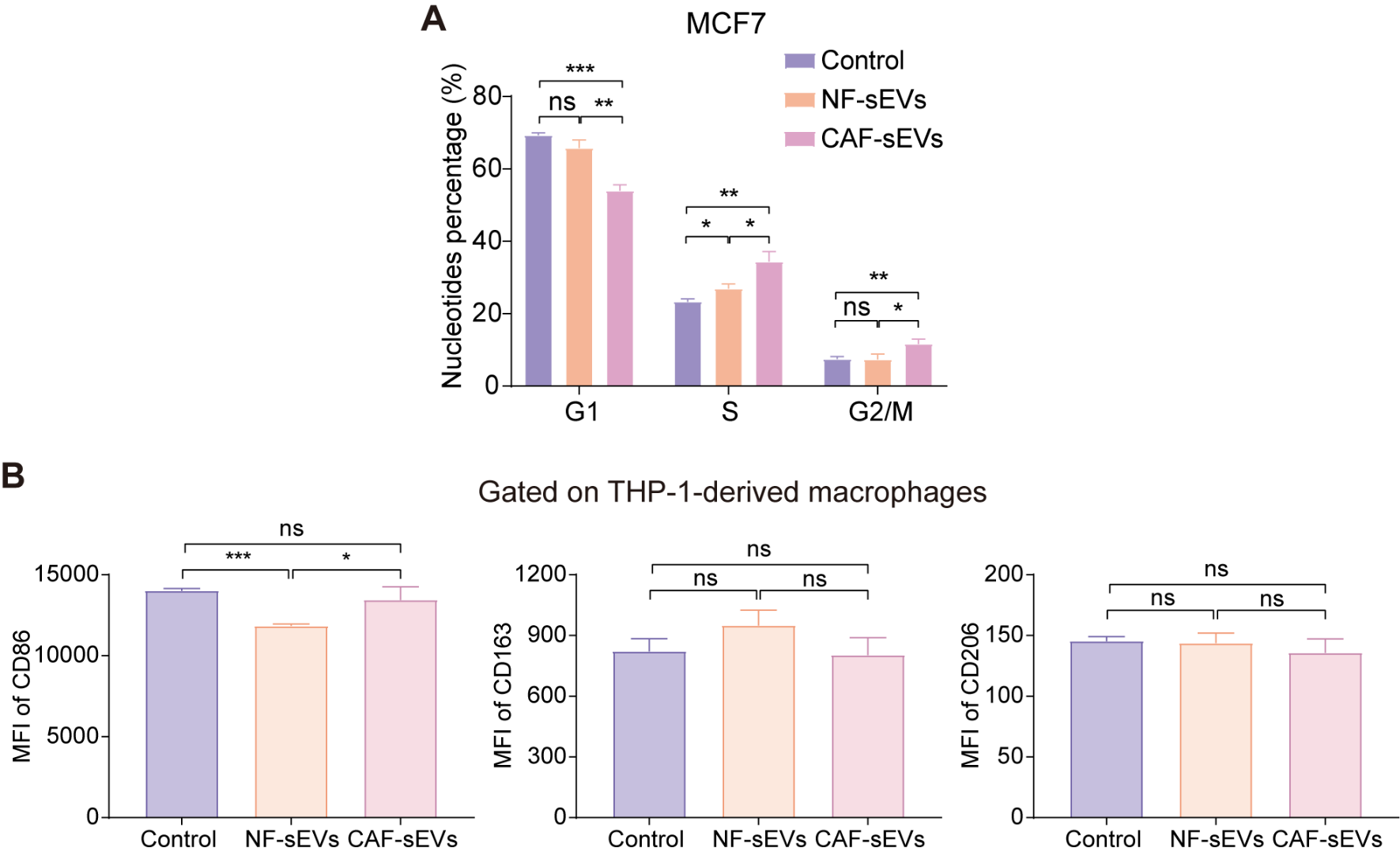
**

**Figure S6. Phenotypic experiments of sEVs derived from CAFs and NFs.** (A) Statistical analysis of the cell cycle performed with propidium iodide DNA staining on MCF7 cells after incubation with equal amounts of CAF-sEVs, NF-sEVs or PBS for 48 h (n = 3). (B) Quantification for the expression levels of CD86, CD163 and CD206 on THP-1-derived macrophages after incubation with equal amounts of CAF-sEVs, NF-sEVs or PBS for 48 h (n = 3). The above graphs show mean ± SD and t test. *p < 0.05, **p < 0.01, ***p < 0.001. ns, not significant.


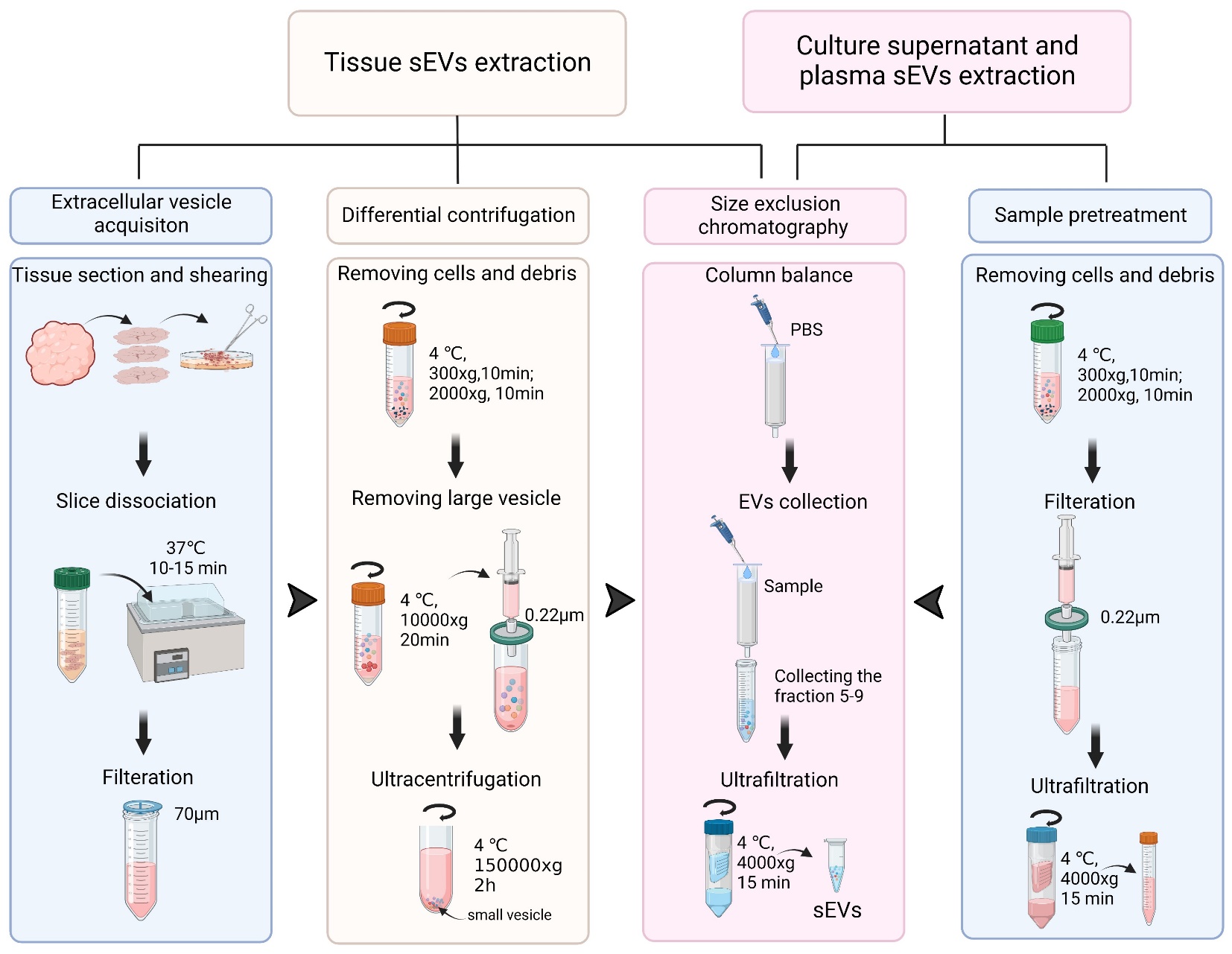


**Figure S7. Schematic diagram of the extraction of sEVs from fresh tissues, culture supernatant and plasma.**

| **Table S1. Overview of involved samples** | | | | | | | | | | | | | |
| --- | --- | --- | --- | --- | --- | --- | --- | --- | --- | --- | --- | --- | --- |
| Patient ID | N-sEVs | T-sEVs | O-sEVs | B-sEVs | CAF-sEVs | Age | Clinical stage | Molecular subtype | ER | PR | HER2 (IHC) | HER2 (FISH) | ki-67 |
| BC#31 |  | T, P |  | T, P |  | 48 | T2N0M0 | Luminal A | 95% | 90% | - |  | 10% |
| BC#32 | P | T | T, P | T, P | T | 63 | T2N0M0 | TNBC | - | - | - |  | 80% |
| BC#33 | T, P | T, P | T, P |  | T | 62 | T1N1M0 | Luminal B | 60% | 5% | ++ | - | 20% |
| BC#35 | T, P | T, P | T, P |  |  | 66 | T1N0M0 | Luminal B | 80% | - | + |  | 15% |
| BC#36 | P | T, P | T, P | T, P | T | 65 | T1N0M0 | Luminal A | 85% | 95% | - |  | 10% |
| BC#40 | T, P | T, P | T, P | T, P |  | 84 | T2N0M0 | Luminal A | 95% | 50% | ++ | - | 20% |
| BC#59 | T, P | T, P | T, P | T, P | T | 48 | T2N0M0 | Luminal B | 85% | 85% | ++ | + | 30% |
| BC#60 | T, P | T, P | T, P | T, P |  | 73 | T2N0M0 | Luminal A | 95% | 95% | - |  | 5% |
| BC#61 | T, P | T, P | T, P |  |  | 44 | T2N3M0 | Luminal A | 90% | 85% | ++ | - | 20% |
| BC#62 | T, P | T, P | T, P | T, P |  | 46 | T1N0M0 | Luminal A | 90% | 90% | - |  | 10% |
| T, transcriptome; P, proteome. | | | | | | | | | | | | | |

| **Table S4. List of cancer-specific sEV mRNAs identified by comparison between groups** | | |
| --- | --- | --- |
| Symbol | p-value | log2FC |
| TESMIN | 0.01158906 | 5.066082797 |
| PRDX1 | 0.021504547 | 1.66479528 |
| HLA-DRA | 0.013574661 | 1.994163944 |
| MMP11 | 0.039347231 | 6.444984221 |
| COX6C | 0.018510901 | 3.059398121 |
| SLC49A4 | 0.043248292 | 6.198785389 |
| FN1 | 0.018510901 | 2.563318472 |
| PRSS8 | 0.043248292 | 3.928137823 |
| CFL1 | 0.033011107 | 0.757533535 |
| CA12 | 0.030752715 | 1.600146638 |
| Human_newGene_1574043 | 0.013574661 | 4.504026623 |
| EVPL | 0.026203048 | 2.697534811 |
| RRM2 | 0.011748347 | 4.775418313 |
| ABRACL | 0.002745322 | 4.745926845 |
| SPOCK2 | 0.0025812 | 7.599794899 |
| GAPT | 0.039327089 | 5.691920423 |
| H3C11 | 0.029696657 | 4.707098538 |
| SERHL2 | 0.020430769 | 5.10093867 |
| MFAP2 | 0.017428424 | 4.584360496 |
| FOXM1 | 0.030295481 | 5.780582405 |
| H2AC11 | 0.04490784 | 2.541077334 |
| PYCARD | 0.045303865 | 2.042861853 |
| SLC25A39 | 0.049545081 | 1.737435029 |
| H3C14 | 0.027149986 | 6.5680736 |
| H2AC14 | 0.018440871 | 4.381378466 |
| ATP6V0B | 0.024804294 | 3.958454523 |
| MMP9 | 0.043248292 | 6.92091137 |
| H3C15 | 0.009528197 | 6.579978757 |
| C6orf136 | 0.039327089 | 3.175758294 |
| Human_newGene_227397 | 0.033638319 | 3.136253391 |
| KRT19 | 0.00678733 | 2.747717031 |
| UNC5B | 0.030295481 | 4.27847961 |
| H2BC15 | 0.010678969 | 4.150858264 |
| HYPK | 0.024989716 | 0.878533179 |
| EPPK1 | 0.018510901 | 2.621980229 |
| ZHX3 | 0.043089264 | 0.945329801 |
| CBX4 | 0.040861608 | 3.923280867 |
| EPCAM | 0.006583061 | 3.971508003 |
